# MAOA‐VNTR genotype affects structural and functional connectivity in distributed brain networks

**DOI:** 10.1002/hbm.24766

**Published:** 2019-08-23

**Authors:** Anais Harneit, Urs Braun, Lena S. Geiger, Zhenxiang Zang, Marina Hakobjan, Marjolein M. J. van Donkelaar, Janina I. Schweiger, Kristina Schwarz, Gabriela Gan, Susanne Erk, Andreas Heinz, Nina Romanczuk‐Seiferth, Stephanie Witt, Marcella Rietschel, Henrik Walter, Barbara Franke, Andreas Meyer‐Lindenberg, Heike Tost

**Affiliations:** ^1^ Department of Psychiatry and Psychotherapy, Central Institute of Mental Health, Medical Faculty Mannheim University of Heidelberg Mannheim Germany; ^2^ Department of Human Genetics Radboud University Medical Center, Donders Institute for Brain, Cognition and Behaviour Nijmegen the Netherlands; ^3^ Language and Genetics Department Max Planck Institute for Psycholinguistics Nijmegen the Netherlands; ^4^ Department of Psychiatry and Psychotherapy Charité ‐ University Medicine Berlin Berlin Germany; ^5^ Department of Genetic Epidemiology in Psychiatry, Central Institute of Mental Health, Medical Faculty Mannheim University of Heidelberg Mannheim Germany; ^6^ Department of Psychiatry Radboud University Medical Center, Donders Institute for Brain, Cognition and Behaviour Nijmegen the Netherlands

**Keywords:** aggression, connectome, imaging genetics, impulsive behavior, monoamine oxidase A gene, multimodal imaging, risk factors

## Abstract

Previous studies have linked the low expression variant of a variable number of tandem repeat polymorphism in the monoamine oxidase A gene (*MAOA‐L*) to the risk for impulsivity and aggression, brain developmental abnormalities, altered cortico‐limbic circuit function, and an exaggerated neural serotonergic tone. However, the neurobiological effects of this variant on human brain network architecture are incompletely understood. We studied healthy individuals and used multimodal neuroimaging (sample size range: 219–284 across modalities) and network‐based statistics (NBS) to probe the specificity of *MAOA‐L*‐related connectomic alterations to cortical‐limbic circuits and the emotion processing domain. We assessed the spatial distribution of affected links across several neuroimaging tasks and data modalities to identify potential alterations in network architecture. Our results revealed a distributed network of node links with a significantly increased connectivity in *MAOA‐L* carriers compared to the carriers of the high expression *(H)* variant. The hyperconnectivity phenotype primarily consisted of between‐lobe (“anisocoupled”) network links and showed a pronounced involvement of frontal‐temporal connections. Hyperconnectivity was observed across functional magnetic resonance imaging (fMRI) of implicit emotion processing (*p*_FWE_ = .037), resting‐state fMRI (*p*_FWE_ = .022), and diffusion tensor imaging (*p*_FWE_ = .044) data, while no effects were seen in fMRI data of another cognitive domain, that is, spatial working memory (*p*_FWE_ = .540). These observations are in line with prior research on the *MAOA‐L* variant and complement these existing data by novel insights into the specificity and spatial distribution of the neurogenetic effects. Our work highlights the value of multimodal network connectomic approaches for imaging genetics.

## 1 INTRODUCTION

Human impulsive and aggressive behaviors are determined by multiple causes involving complex interactions between genetic and environmental risk factors, such as *Monoamine Oxidase A* (*MAOA*) genotype and early life events (Byrd & Manuck, 2014; Caspi et al., 2002). MAOA is a key mitochondrial enzyme involved in serotonin and norepinephrine catabolism, which is important during brain development as well as brain function (Shih, Chen, & Ridd, 1999). An upstream variable number of tandem repeat (uVNTR) polymorphism in the *MAOA* promoter region, which is common in the population, also has a strong impact on transcriptional efficacy, with high enzyme expression in carriers of 3.5 or 4 repeats (*MAOA‐H*) and low enzyme expression in carriers of 2, 3, or 5 repeats (*MAOA‐L*; Guo, Ou, Roettger, & Shih, 2008; Sabol, Hu, & Hamer, 1998). Further evidence links *MAOA‐L* to antisocial traits and behaviors (Mertins, Schote, Hoffeld, Griessmair, & Meyer, 2011; Williams et al., 2009), and to psychiatric disorders with impulsive features including attention deficit hyperactivity disorder (Manor et al., 2002) and alcohol dependence (Contini, Marques, Garcia, Hutz, & Bau, 2006).

The neurobiological mechanisms by which *MAOA‐L* impacts aggression are incompletely understood, with the most consistent evidence converging on cortico‐limbic regions. In particular, increased amygdala reactivity to negative emotional stimuli (Lee & Ham, 2008; Meyer‐Lindenberg et al., 2006), reduced activations in medial frontal and anterior cingulate cortex during inhibitory control (Meyer‐Lindenberg et al., 2006; Passamonti et al., 2008), and reduced cortico‐limbic gray matter volume (Cerasa, Gioia, Fera, et al., 2008; Cerasa, Gioia, Labate, et al., 2008; Meyer‐Lindenberg et al., 2006) have been repeatedly detected. Moreover, functional connectivity analyses revealed increased coupling between amygdala and higher order areas in the ventromedial prefrontal (Buckholtz et al., 2008) and anterior cingulate (Denson, Dobson‐Stone, Ronay, von Hippel, & Schira, 2014) cortices (Denson et al., 2014) in *MAOA‐L* carriers. Together, these data suggest that the low‐activity allele of *MAOA* facilitates alterations in cortico‐limbic circuits critical for negative emotion regulation and inhibitory control, likely through excessive serotonergic signaling during vulnerable periods of brain development (Cases et al., 1995). Environmental factors like early life adversity and drug intake might exacerbate its impact on monoaminergic neurotransmission (Heinz, Beck, Meyer‐Lindenberg, Sterzer, & Heinz, 2011; Meyer‐Lindenberg et al., 2006). However, most imaging genetics studies on *MAOA* focused on specific brain functional domains, a limited set of neural regions and/or a single neuroimaging data modality, making a more comprehensive understanding of the neural connectomic effects difficult (Klein, van Donkelaar, Verhoef, & Franke, 2017).

Several lines of evidence suggest more widespread effects of *MAOA* on neural structural and functional network architecture. First, positron emission tomography (PET) and postmortem studies revealed a broad topological distribution of MAOA binding potentials and mRNA expression levels across the human brain (Komorowski et al., 2017; Tong et al., 2013). Second, the encoded enzyme is a central modulator of stem cell neural differentiation and neural circuit segregation during early brain development (Ou, Chen, & Shih, 2006; Wang, Chen, Ying, Li, & Shih, 2011). In MAOA‐deficient mice, the ensuing disturbances in brain maturation are well‐established and include distributed neural regions and a range of behavioral alterations (Bortolato et al., 2013; Cheng et al., 2010; Upton et al., 1999). These data suggest that a functional change in the gene can lead to distributed network effects, which extend across brain functional domains and neuroimaging data modalities.

Given this evidence, we sought to extend prior research on *MAOA* by studying the human brain connectome using multimodal neuroimaging and a regionally unconstrained whole‐brain network‐based analysis approach. We analyzed healthy individuals to prevent confounding by the presence of brain disorders. Specifically, we aimed to examine whether functional connectomic alterations during negative emotion processing (a) are limited to cortico‐limbic circuits, (b) are specific to the emotion processing domain, and (c) go along with comparable structural alterations in neural network architecture. In supplementary analyses, we further assessed the spatial distribution of affected links across neuroimaging tasks and modalities and explored the sub‐networks for potential associations with emotion regulation ability and recent stressful life events. Based on the existing connectivity literature (Buckholtz et al., 2008; Denson et al., 2014), we posited a regionally distributed pattern of “hyperconnected” link clusters in the *MAOA‐L* carriers, a pattern we expected to extend across brain functional domains and to include structural connectomic alterations.

## 2 MATERIALS AND METHODS

### 2.1 Participants

We included data from 219 to 284 healthy adult participants per neuroimaging modality. Individuals were of European ancestry and were recruited from the general population at three German sites (Mannheim, Berlin, Bonn). General exclusion criteria were a lifetime history of significant general medical, psychiatric, or neurological illness, the presence of a first‐degree relative with a history of psychiatric illness, prior drug or alcohol abuse, and head trauma. All subjects provided written informed consent for protocols approved by the institutional ethical review boards of the Universities of Heidelberg, Bonn, and Berlin. Demographic and clinical information was available for all individuals. Data on the preferred tendency to regulate emotions (as measured by the Emotion Regulation Questionnaire, ERQ) was available in a subset of 259 individuals (Gross & John, 2003). Data on the extent of stressful life events in the preceding 2 years (as measured by the social readjustment rating scale, SRRS) was available in a subset of 260 individuals (Holmes & Rahe, 1967).

### 2.2 *MAOA* genotyping

The Supporting Information “Methods” section provides details on the genotyping procedures and detected allele frequencies (Table Data S1). Since the *MAOA* gene is located on the X chromosome, males are hemizygous carriers of either one *L* or *H* allele. Women carry two alleles and can thus be heterozygous, although one of the two alleles is (fully or incompletely) silenced by random X chromosome inactivation (Berletch, Yang, Xu, Carrel, & Disteche, 2011). We addressed this ambiguity by excluding all *MAOA* heterozygous females from subsequent analysis. Sex distribution differed significantly between *MAOA* genotype groups in our study cohort (*p* < .001). We addressed this issue by adding sex as a covariate in all statistical analyses. There were no additional significant differences in demographic, psychological, and fMRI performance data between genotype groups (all *p*‐values >.27 Table 1).

**Table 1:** Sample characteristics stratified by *MAOA* genotype

	*H* allele carriers	*L* allele carriers	*p*‐value
*Demographics*			
Age (year)	33.69 ± 10.00	33.18 ± 9.53	.69
Sex (males/females)	107 / 89	65 / 15	<.001
Site (Berlin/Bonn/Mannheim)	50 / 81 / 65	24 / 33 / 23	.68
Education (years), mean ± *SD*	15.35 ± 2.48	15.69 ± 2.71	.32
Handedness (right/left/both)	174 / 17 / 4	73 / 6 / 1	.85
*Questionnaires*			
ERQ‐suppression	14.13 ± 4.99	13.30 ± 4.78	.91
ERQ‐reappraisal	27.12 ± 6.57	27.49 ± 6.87	.62
SRRS	273.05 ± 190.77	321.11 ± 203.93	.76
*fMRI task performance*			
Faces condition (% correct)	98.74 ± 2.84	97.97 ± 4.89	.32
Forms condition (% correct)	97.28 ± 4.07	96.64 ± 5.02	.55
2‐back condition (% correct)	75.91 ± 21.47	73.75 ± 21.19	.40
0‐back condition (% correct)	98.41 ± 5.93	98.40 ± 5.39	.94
*MRI data quality*			
Faces task: Mean frame‐wise displacement (mm)	0.16 ± 0.08	0.17 ± 0.08	.99
*n*‐back task: Mean frame‐wise displacement (mm)	0.14 ± 0.006	0.13 ± 0.06	.27
Resting task: Mean frame‐wise displacement (mm)	0.17 ± 0.06	0.16 ± 0.05	.48
DTI: Mean frame‐wise displacement (mm)	0.86 ± 0.33	0.81 ± 0.32	.32

Abbreviations: DTI, diffusion tensor imaging, ERQ, Emotion Regulation Questionnaire (calculated as the sum of contributing subscale item scores), SRRS, social readjustment rating scale (calculated from the assessment of life events in the last 2 years). Categorical variables are reported as numbers of cases, continuous variables are reported as mean and standard deviation (*SD*).

### 2.3 MRI data acquisition

We collected multimodal magnetic resonance imaging (MRI) data with three identical Siemens 3‐T scanner systems (Siemens Trio, Erlangen, Germany) located at the three sites. Table S2 provides an overview of participant numbers and characteristics for each data modality. In brief, high quality imaging data was available in a subset of 247 individuals for fMRI emotion processing, 219 individuals for fMRI resting‐state, 254 individuals for fMRI working memory, and 284 individuals for DTI structural data. The fMRI and high‐resolution T1‐weighted images were acquired with the same protocol; DTI data were acquired with four slightly different protocols. Further details are provided in Supporting Information “Methods” section.

### 2.4 fMRI paradigms

We used three well‐established fMRI tasks probing implicit emotion processing (emotional face matching task), resting‐state (rs‐fMRI) and working memory (*n*‐back task), as previously described in detail (Callicott et al., 2004; Cao et al., 2014; Hariri et al., 2002). All participants were thoroughly trained on the tasks prior to the scan. Further details on the tasks are provided in Supporting Information “Methods” section.

### 2.5 Functional MRI data processing and connectome construction

Functional networks were constructed following previously published procedures (Cao et al., 2014, 2016; Zang et al., 2018) using SPM8 and MATLAB. In short, data preprocessing included realignment to the mean image of the time series, slice time correction, spatial normalization to the Montreal Neurological Institute (MNI) EPI template, and smoothing with an 8 mm full‐width at half‐maximum (FWHM) Gaussian kernel. For each participant and fMRI task, we then extracted the mean time series from the 116 brain regions (or nodes) defined by the automated anatomical labeling (AAL) atlas (Tzourio‐Mazoyer et al., 2002). From the node time series, we regressed out white matter and cerebrospinal fluid signals, the mean effect of task conditions (active tasks only), and the six head motion parameters from the realignment step. The resulting residual time series were temporally filtered using a 0.008–0.1 Hz bandpass filter for the resting‐state data and a highpass filter (cut‐off 128 s) for the face matching and *n*‐back data. As functional connectivity data can be severely affected by head micromovements, we used in‐house software to estimate frame‐wise displacement (FD) for all functional data and scrubbed all data frames with a FD > .5 mm and interpolated the missing frames using a B‐spline interpolation. Subjects with more than 10% affected data frames were excluded from the analysis. We then calculated pairwise Pearson correlation coefficients between each pair of nodes resulting in a 116 × 116 connectivity matrix for each subject and functional data type.

### 2.6 Structural MRI data processing and connectome construction

DTI data preprocessing was performed with standard routines implemented in the software package FSL (Smith et al., 2004) including the following steps: correction of the diffusion images for head motion and eddy currents by affine registration to a reference (b0) image, extraction of nonbrain tissues, and linear diffusion tensor fitting. After estimation of the diffusion tensor, we performed deterministic whole‐brain fiber tracking as implemented in DSI Studio using a modified FACT algorithm (Yeh, Verstynen, Wang, Fernandez‐Miranda, & Tseng, 2013). For the construction of structural connectivity matrices, we initiated 1,000,000 streamlines for each participant. Streamlines with a length of less than 10 mm were removed. The total number of successful streamlines between each pair of nodes defined by the AAL atlas was then used as estimates of structural connectivity, resulting in a 116 × 116 connectivity matrix for each subject.

### 2.7 Data analysis and statistical inference

We used network‐based statistics (NBS) to identify clusters of functional and structural links significantly differing between *MAOA* genotype groups. NBS is a well‐established method for controlling cluster‐level family‐wise error (FWE) rates for link‐wise comparisons while providing an increased power compared to mass‐univariate tests on individual links (Zalesky, Fornito, & Bullmore, 2010). For comparability between data modalities, we used the following identical analysis parameters for all fMRI and DTI data: We identified suprathreshold links and sets of connected link clusters using ANOVA models with genotype as between‐subjects factor (*MAOA* high vs. *MAOA* low) and the covariates age, sex, data acquisition site, and sequence protocol (initial threshold: *p* ≤ .005, uncorrected). The significance of the link clusters was assessed by performing 5,000 permutations, in which subjects were randomly reassigned to genotype groups, and the maximal extent of the identified cluster was recalculated. The FWE‐corrected *p*‐value for the identified clusters was determined by the proportion of cluster sizes in the permutation distribution that was larger than the cluster sizes of the observed group difference. This procedure was applied to all imaging modalities.

### 2.8 Supplemental exploratory analyses

To further quantify the spatial distribution of *MAOA*‐affected node connections across the brain and descriptively compare the outcome between different neuroimaging tasks and modalities, we post hoc quantified and illustrated the percentage of significant “isocoupled” versus “anisocoupled” links, that is, connections between neural nodes within the same versus between different major brain subdivisions as defined by the six supraordinate labels of the AAL atlas (i.e., frontal, parietal, occipital, temporal, cingulate, and subcortical regions). Moreover, since *MAOA* is located on the X chromosome, and the gene is of interest for impulsivity and aggression, we further explored the potential impact of sex on the identified *MAOA*‐associated cluster links by testing the mean cluster connectivity estimates for potential genotype by sex interaction effects across all imaging modalities. In addition, we tested whether the reported effects of *MAOA* genotype on cluster connectivity remains stable in subsamples with sex‐matched genotype groups and in separate analyses of males and females, respectively. Details on the participant numbers of the sex‐matched supplemental analyses are provided in Table S3.

### 2.9 Relationship to emotion regulation

In addition, since *MAOA* genotype has been linked to dysfunctional responses to negative emotional events (Kim‐Cohen et al., 2006), we explored whether the connectivity estimates of the identified *MAOA*‐associated sub‐networks across tasks and modalities related to the tendency of individuals to employ a maladaptive emotion regulation strategy. For this, we first quantified the mean sub‐network connectivity estimates for the *MAOA*‐associated networks and the scores for ERQ subscales, representing two emotion regulation strategies, “cognitive reappraisal” (higher values indicate psychiatric resilience) and “expressive suppression” (higher values indicate psychiatric risk) for each individual and subsequently calculated Pearson correlation coefficients, controlling for age, sex, and data acquisition site. We used analogous procedures to test sub‐networks across tasks and modalities for potential associations with recent stressful life events (SRRS total scores).

## 3 RESULTS

### 3.1 Functional network analyses

**Figure 1:**
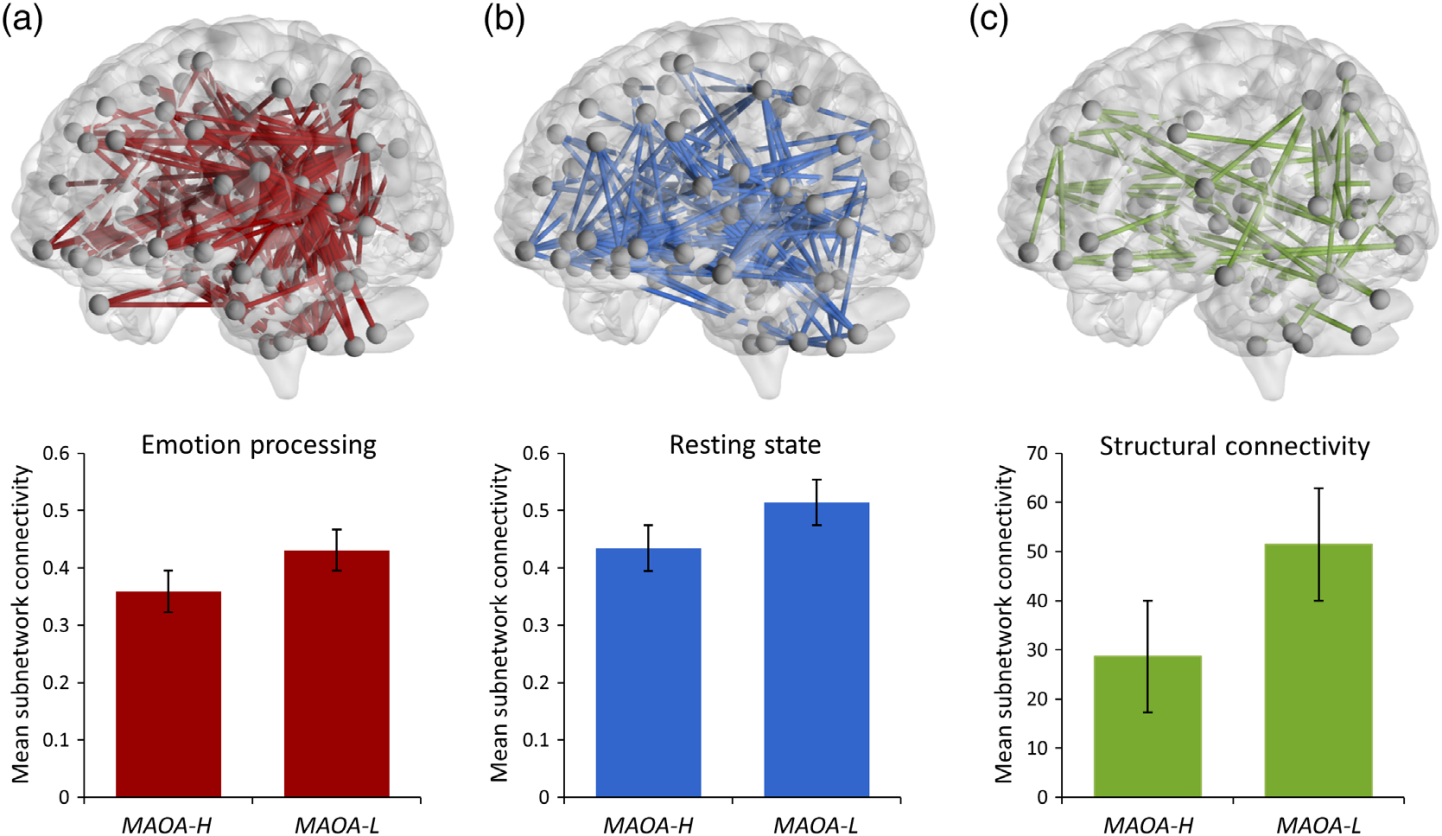
Illustration of the whole‐brain spatial distribution (upper panels) and mean connectivity values of the identified *MAOA*‐affected brain sub‐networks stratified by genotype (lower panels) demonstrating significantly increased connectivity in *MAOA‐L* allele carriers across (a) emotion processing (*p*_FWE_ = .037, 248 links), (b) resting‐state (*p*_FWE_ = .022, 176 links), and (c) diffusion tensor imaging/structural connectivity (*p*_FWE_ = 0.044, 48 links) data. Bars indicate mean values, error bars indicate standard errors [Color figure can be viewed at http://wileyonlinelibrary.com]

In our starting hypothesis to this work, we posited that alterations in the neural functional connectivity in carriers of the risk‐associated low expression (*L*) variant in *MAOA* would involve, but not necessarily be exclusive to, frontal‐temporal neural circuits during implicit emotion processing. Consistent with this, our NBS analysis of the emotional face matching task data identified a distributed cluster of node links with a significantly increased functional connectivity in *L* allele carriers compared to *H* allele carriers (*p*_FWE_ = .037, Figure 1a). The identified sub‐network included, but was not limited to, frontal‐temporal areas and consisted of a total of 82 nodes that were connected by 248 links (or edges). To assess the potential specificity of the identified *MAOA*‐associated functional network effects for emotion processing, we assessed *MAOA* genotype effects on the functional connectivity of node links also during resting‐state and working memory performance. For resting‐state fMRI, our NBS analysis detected a comparably distributed cluster of 176 links interconnecting 82 nodes with a significant increase in functional connectivity in *MAOA‐L* allele carriers compared to *MAOA‐H* allele carriers (*p*_FWE_ = .022, Figure 1b). In contrast, the NBS analysis of the working memory data yielded a null finding (*p*_FWE_ = .540).

### 3.2 Structural network analysis

The NBS analysis of DTI data identified a brain sub‐network with a significant increase in structural connectivity in the *MAOA*‐*L* allele carriers compared to *H* allele carriers (*p*_FWE_ = .044, Figure 1c). The identified cluster consisted of 48 links interconnecting 43 nodes. The cluster was distributed comparably to the *MAOA*‐related functional links, although smaller in extent. For all imaging modalities, details on the identified *MAOA*‐associated cluster links are provided in Tables S4–S6. For illustration purposes, the tables include the corresponding anatomical location of nodes in AAL standard space and highlight the prominent role of frontal lobe connections (bolded) and “isocoupled” links (italicized) of the respective anatomical labels. Moreover, the top 10% of the most significant *MAOA*‐related nodes and interconnecting links for each modality are highlighted in red to facilitate the assessment of important anatomical contributors to the respective *MAOA*‐related cluster findings.

### 3.3 Supplemental exploratory analyses

**Figure 2:**
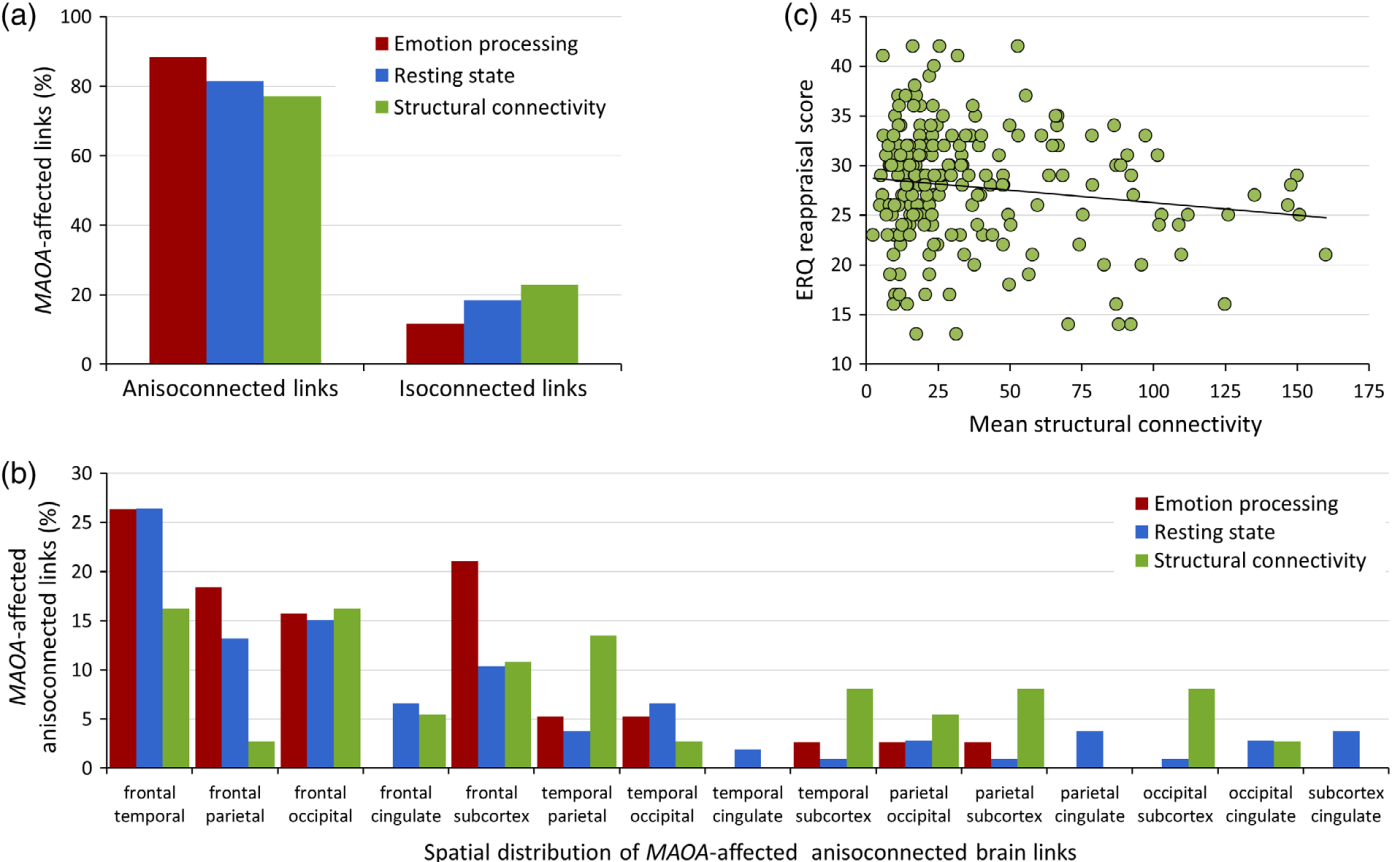
(a) Percentage (*y* axis) of *MAOA*‐affected connections across tasks and modalities stratified by (*x* axis) the anisocoupled versus isocoupled nature of node links (see text for definition of terms). (b) Percentage (*y* axis) and spatial distribution (*x* axis) of *MAOA*‐affected anisocoupled brain links across tasks and modalities. (c) Illustration of the association (*p* = .041, *r* = −0.37) between Emotion Regulation Questionnaire (ERQ) cognitive reappraisal scores (*y* axis) and the mean connectivity of the *MAOA*‐affected structural network (quantified by the mean of the successful diffusion tensor imaging [DTI] fiber tracking streamlines) [Color figure can be viewed at http://wileyonlinelibrary.com]

We detected no significant interaction between *MAOA* genotype and sex on mean network connectivity scores in any MRI modality (all *p‐*values >.88). Post hoc regional quantification of the *MAOA*‐significant links suggested a clear commonality across tasks and modalities in the form of a higher ratio of affected connections between neural nodes from different major AAL brain sections (77–88% anisocoupled links, as compared to 12–23% isocoupled links relating different nodes from the same major brain subdivision, Figure 2a). In addition, across all *MAOA*‐significant tasks and modalities, a relatively high involvement of links interconnecting the frontal lobe with temporal, occipital, and subcortical regions was apparent. Obvious modality‐specific patterns did not arise; the regional distribution of altered connections appeared to be the most unspecific in the structural data and included a higher proportion of *MAOA*‐associated links interconnecting subcortical structures (Figure 2b). Notably, the reported effects of *MAOA* genotype on cluster connectivity remained stable in our follow‐up analyses in subsamples with sex‐matched genotype groups across modalities (all *p*‐values <.01). Similarly, in the sex‐matched subsamples (Table S3), the association of *MAOA* genotype with cluster connectivity estimates remained significant for both genders and across modalities when we analyzed males and females separately (all *p*‐values <.04).

### 3.4 Relationship to emotion regulation

Our exploratory analyses on the relationship between the *MAOA*‐associated sub‐network parameters and emotion regulation strategy showed a significant negative correlation of the connectivity of the structural network with the ERQ cognitive reappraisal subscale (*p* = .041, *r* = −0.37, Figure 2c). No significant correlations of the structural network with the connectivity parameters of the functional sub‐networks or the ERQ expressive suppression subscale were seen (all *p*‐values >.335). We did not detect any significant associations between *MAOA* genotype and stressful life events as assessed with the Social Readjustment Rating Scale scores (all *p*‐values >.18). *MAOA* genotype was not associated with any of the ERQ subscale scores (all *p*‐values >.16).

## 4 DISCUSSION

In this study, we aimed to extend the current understanding of the neurogenetic risk architecture of brain circuits underlying impulsivity and aggression using multimodal neuroimaging and whole‐brain connectomic methods in healthy humans stratified by *MAOA* genotype. Our analyses identified several structural and functional network alterations in the carriers of the risk‐associated low expressing variant.

First, we identified a functional connectomic phenotype during implicit emotion processing manifesting as a regionally distributed set of hyperconnected brain nodes in individuals with the *MAOA‐L* genotype. Among the affected node links, we classified more than 80% as edges affecting functional interactions between distant major anatomical subdivisions of the brain (“anisocoupled links”). These observations are consistent with the interpretation that the low expressing *MAOA* variant preferentially affects long‐range connections, which likely serve to control and integrate information across different brain areas with more specialized neural functions enabling a rich set of brain dynamics (Alexander‐Bloch et al., 2013; Betzel & Bassett, 2018). From the affected long‐range integrative links, nearly half (47%) functionally coupled the frontal cortex to the temporal lobe and subcortical regions. This finding is in line with the established view that *MAOA‐L* mainly impacts prefrontal circuits with a top‐down regulatory influence on subordinate neural regions generating evolutionarily conserved physiological responses (Dorfman, Meyer‐Lindenberg, & Buckholtz, 2014). As in prior connectivity studies on *MAOA‐L*, the detected connectomic alterations during the processing of emotionally charged information manifested as a significant increase in functional coupling (Buckholtz et al., 2008; Denson et al., 2014). This alteration has been previously interpreted as a neurogenetic disruption of prefrontal regulatory circuits predisposing *MAOA‐L* individuals to exaggerated and less controlled responses charged with impulsive arousal and negative emotions. With respect to our first research question, we thus conclude that the identified *MAOA*‐related alterations in functional connectivity during negative emotion processing are consistent with the idea of a pronounced, but not exclusive, involvement of prefrontal‐limbic circuits. Taken together, our emotion processing data are well in line with the existing imaging genetics literature and extend our current understanding of *MAOA* by providing novel connectomic insights arising from a whole‐brain network approach.

Second, we probed the detected network connectivity alterations for specificity to the emotion‐processing domain by analysis of resting‐state and working memory data. In general, the study of the human brain at rest is valuable since connected brain networks display coordinated low‐frequency fluctuations in the absence of external stimulation, thereby providing insights into the basic functional architecture of interacting networks. Similar to the emotion‐processing domain, our resting‐state analysis identified a regionally distributed set of hyperconnected links in *MAOA‐L* carriers, which mostly related nodes from distant anatomical subdivisions of the brain. More than two thirds (69%) of the altered links connected the frontal cortex to other cortical and subcortical targets. With respect to our second research question, this indicates that the assumed neurogenetic modulation of prefrontal regulatory circuits related to *MAOA‐L* is likely not limited to emotionally charged tasks, but rather reflective of an alteration of basic functional architecture of the brain. To date, resting‐state fMRI studies on *MAOA* are very sparse, making the integration of our findings challenging. However, a prior exploratory resting‐state study using independent component analysis methods identified increased connectivity of several prefrontal and temporal areas in the risk‐associated *MAOA‐L* carriers (Clemens et al., 2015), which is in line with our findings, albeit obvious differences in the employed approach.

The assumption of a more profound impact of the *MAOA‐L* genotype on human brain architecture is further supported by our structural network findings. Diffusion‐based research on this genetic variant is lacking to date, but our deterministic tracking of white matter projections revealed first evidence for a distributed pattern of hyperconnected node links in *MAOA‐L* carriers, with large proportion of the affected connections (40%) mapping to frontal “anisocoupled” cortical connections (Table S5). With respect to our third research question, our data thus indicate that the assumed predisposition for an alteration in human neural network architecture does include hard‐wired anatomical links, most notably those pertaining to long‐range connections of prefrontal regulatory circuits, which likely regulate and integrate bottom‐up input from other brain areas.

We did not observe *MAOA‐L*‐related network alterations during working memory performance, which appears to contradict our interpretation of widespread effects of the variant on network architecture. Working memory tasks typically require coordinated frontal‐parietal functions, and only about 3% of *MAOA*‐sensitive structural links pertained to frontal‐parietal connections (Figure 2). Prior evidence on the effects of *MAOA* on working memory function is conflicting, with one study reporting a positive association finding in high‐load cognitive conditions (Cerasa, Gioia, Fera, et al., 2008; Cerasa, Gioia, Labate, et al., 2008) and several negative findings (Barnett, Xu, Heron, Goldman, & Jones, 2011; Dumontheil et al., 2014; Soderqvist, Matsson, Peyrard‐Janvid, Kere, & Klingberg, 2014). Importantly, the fMRI *n*‐back task employed in this study challenges executive neural networks but is comparatively simple, and the load on additional regulatory resources for impulsive and emotional responses is low. Thus, it may well be possible that the neurogenetic consequences of *MAOA* genotype impact distributed regulatory networks involved in top‐down prefrontal regulatory control, including alterations in hard‐wired anatomical links, but that these differences are not focused on frontal‐parietal networks. Therefore, the potentially resulting challenges for working memory may remain masked unless additional functional requirements related to high cognitive load including frustration and emotion regulation requirements are challenged (Cerasa, Gioia, Fera, et al., 2008; Cerasa, Gioia, Labate, et al., 2008). Further research is needed to corroborate this hypothesis.

Our findings support the notion of *MAOA*‐related genetic associations in cortico‐limbic circuits, and structural and functional alterations in these circuits have been linked to maladaptive emotion regulation. For example, *MAOA* has been previously related to a number of psychiatric risk‐associated behaviors with deficient emotion regulatory capacity including impulsivity (Chester et al., 2015; Meyer‐Lindenberg et al., 2006) and aggression (Brunner, Nelen, Breakefield, Ropers, & van Oost, 1993; Eisenberger, Way, Taylor, Welch, & Lieberman, 2007; Gallardo‐Pujol, Andres‐Pueyo, & Maydeu‐Olivares, 2013; Kuepper, Grant, Wielpuetz, & Hennig, 2013; McDermott, Tingley, Cowden, Frazzetto, & Johnson, 2009; Raine, 2008) as well as clinical conditions such as depression (Fan et al., 2010; Liu, Huang, Luo, Wu, & Li, 2016) and attention deficit hyperactivity disorder (ADHD; Das et al., 2006; Hwang, Lim, Kwon, & Jin, 2018). Recent lines of evidence suggest that these behaviors and disorders are shaped by complex gene–environment interactions (Melas et al., 2013; Palumbo, Mariotti, Iofrida, & Pellegrini, 2018; Shumay, Logan, Volkow, & Fowler, 2012) that may plausibly relate to epigenetically mediated effects on brain network structure and function. Thus, the extension of our imaging genetics approach to *MAOA*‐related epigenetic network associations and the inclusion of psychiatric populations in future work appears valuable.

Importantly, there are several methodological constraints of our study meriting further consideration. Firstly, as in virtually all neuroimaging studies in humans, the analyzed system level metrics are indirect in nature and reflect only to some extent the microscale biological features of neural structure and function. Secondly, since we aimed for a common reference framework, we used the same anatomical atlas for fMRI and DTI to be able to compare the identified *MAOA*‐sensitive structural and functional brain networks across data modalities. However, the direct comparison of anatomical and functional connections is limited by the inherently different nature of the respective connectivity metrics. Specifically, while our DTI networks represent deterministic reconstructions of putative structural fiber tracts, our functional networks represent the stochastic association between the BOLD‐fluctuations of brain regions. Moreover, deterministic structural networks are typically sparse, with only 2–10% of the initiated streamlines reaching their target (Betzel & Bassett, 2018; Hagmann et al., 2008), while the functional networks are, in principle, fully connected and show a complex structure of interdependent links, based on the correlative methods by which they are constructed. Given these differences, we restricted ourselves to a descriptive comparison of modalities as suggested by previous studies. Finally, although the identified association between the connectivity estimates of the identified *MAOA*‐sensitive structural network and the relative lack of a protective emotion regulation strategy is plausible in the context of the studied variant, this observation comes from an exploratory analysis and requires replication. We found it nonetheless useful to report this finding to aid the formation of specific hypotheses in future studies and encourage replication attempts.

In conclusion, to our knowledge, this is the first whole‐brain multimodal connectomic study on the effects of *MAOA‐L* genotype in healthy humans. Our data suggest that the low expression variant facilitates distributed alterations in network architecture across imaging modalities and tasks, which preferentially involve longer‐range links connecting the prefrontal lobe with temporal, occipital, and subcortical regions. The identified multimodal hyperconnectivity profiles of the risk‐associated low expression variant include the structural and functional connectome, although functional networks during low to medium working memory load appear to be spared. Our findings are well in line with prior studies suggesting *MAOA*‐related genetic alterations in cortico‐limbic circuits critical for negative emotion regulation, which are believed to result from excessive serotonergic signaling during vulnerable periods of brain development; we extend these data by novel multimodal whole‐brain connectomic insights.

## DISCLOSURE

A.M.‐L. has received consultant fees from American Association for the Advancement of Science, Atheneum Partners, Blueprint Partnership, Boehringer Ingelheim, Daimler und Benz Stiftung, Elsevier, F. Hoffmann‐La Roche, ICARE Schizophrenia, K. G. Jebsen Foundation, L.E.K Consulting, Lundbeck International Foundation (LINF), R. Adamczak, Roche Pharma, Science Foundation, Sumitomo Dainippon Pharma, Synapsis Foundation—Alzheimer Research Switzerland, System Analytics, and has received lectures fees including travel fees from Boehringer Ingelheim, Fama Public Relations, Institut d'investigacions Biomèdiques August Pi i Sunyer (IDIBAPS), Janssen‐Cilag, Klinikum Christophsbad, Göppingen, Lilly Deutschland, Luzerner Psychiatrie, LVR Klinikum Düsseldorf, LWL Psychiatrie Verbund Westfalen‐Lippe, Otsuka Pharmaceuticals, Reunions i Ciencia S. L., Spanish Society of Psychiatry, Südwestrundfunk Fernsehen, Stern TV, and Vitos Klinikum Kurhessen. B.F. has received educational speaking grants from Medice and Shire. The remaining authors have nothing to disclose.

## Supporting information


**Data S1**: Supporting information
