# Supplementary material for: MAOA‐VNTR genotype affects structural and functional connectivity in distributed brain networks
Source: Hum Brain Mapp. 2019 Aug 23;40(18):5202–12. doi: 10.1002/hbm.24766 (PMC6864897; doi:10.1002/hbm.24766)
Supplement: Supplementary file 1 — Data S1: Supporting information [file HBM-40-5202-s001.docx]

**Supplements**

**Materials and Methods**

Genotyping procedures

We genotyped a total of 504 individuals using standard methods to extract genomic DNA from lymphoblastoid cell lines. The MAOA 30 bp repeat polymorphism was genotyped using 30 ng genomic DNA as template. PCR was performed with 1x AmpliTaq Gold® 360 Master Mix (Life Technologies) and 0.33 mM fluorescently labeled forward primer (FAM, VIC, PET or NED - 5’- ACAGCCTGACCGTGGAGAAG-3′) and reverse primer (5′- GAACGGACGCTCCATTCGGA -3′) in a total volume of 7,5 µl. Amplification was performed using the following protocol: 95⁰C for 10 min followed by 35 cycles of denaturation for 30 s at 95⁰C, 30 s annealing at 60⁰C, and primer extension at 72⁰C for 1 min, followed by a final extension at 72⁰C for 10 min. The product of the amplification was diluted 1:20 in H_2_O.

Determination of the length of the alleles was performed by direct fragment length analysis on an automated capillary sequencer (ABI3730, Applied Biosystems, Nieuwerkerk a/d Ijssel, The Netherlands) using standard conditions (1 µl of the diluted PCR product together with 9.7 µl formamide and 0.3 µl GeneScan-600 Liz Size StandaardTM (Applied Biosystems, Nieuwerkerk aan de IJssel, the Netherlands)). Results were analyzed with Genemapper version 4.0 (Applied Biosystems). Generally, the MAOA genotyping assay has been validated earlier, and 5% blanks as well as duplicates between plates were taken along as quality controls during genotyping.

Genotyping was performed in a JCI-accredited laboratory at the Department of Human Genetics of the Radboud University Medical Center in Nijmegen.

We categorized 2, 3 and 5 copies of the repeat sequence as *MAOA-L* and 3.5. or 4 copies as *MAOA-H (*[*Guo, Ou, Roettger, & Shih, 2008*](#_ENREF_3)*;* [*Sabol, Hu, & Hamer, 1998*](#_ENREF_5)*)*. The exact allele frequencies are given in ***Supplementary Table 1***. As detailed in the main manuscript, we excluded all *MAOA* female heterozygotes from subsequent analysis.

MRI data acquisition

For fMRI data acquisition, we used gradient-recalled echo-planar imaging (GRE-EPI) sequences with the following parameters: TR 2000ms, TE 30ms, 28 oblique slices per volume, 4mm slice thickness, 1mm slice distance, 80° flip angle, 192mm FOV, and 64 × 64 matrix. For DTI data acquisition, we used spin echo EPI sequences with the following parameters: 1) Mannheim: TR 14000ms, TE 86ms, 2mm slice thickness, 60 non-collinear directions, b-value 1000 s/mm^2^, 1 b0 image, FOV 256 mm; 2) Bonn: TR 12000ms, TE 100ms, 1.7mm slice thickness, 60 non-colinear directions, b-value 1000 s/mm^2^, 11 b0 images, FOV 220mm; 3) Berlin: TR 8200mm, TE 92ms, 2.3mm slice thickness, 64 non-colinear directions, b-value 1200 s/mm^2^, 7 b0 images, FOV 220mm. Since good quality DTI data were only available for a subset of 211 individuals we added 73 DTI data sets from an additional adult cohort of healthy volunteers to obtain comparable sample sizes for the functional and the structural connectivity analyses. The spin echo EPI sequence of the additional Mannheim cohort had the following parameters: TR 1400ms, TE 93ms, 1.7mm slice thickness, 60 non-collinear directions, b-value 1500 s/mm^2^, 3 b0 images, FOV 220mm.

**Supplementary Table 1: Genotype frequencies**

|  | *L* hemi-/ homozygotes | | | *H* hemi-/ homozygotes | | | | *L/H* heterozygotes | | | | | |
| --- | --- | --- | --- | --- | --- | --- | --- | --- | --- | --- | --- | --- | --- |
|  |  | | |  | | | |  | | | | | |
| Females | 2r/2r | 3r/3r | 5r/5r | 3.5r/4r | 3.5r/3.5r | | 4r/4r | 2r/3.5r | 3r/3.5r | 5r/3.5r | 2r/4r | 3r/4r | 5r/4r |
|  | 1 | 27 | 1 | 5 | 0 | | 129 | 0 | 2 | 0 | 0 | 104 | 4 |
|  |  | | |  |  | |  |  | | | | | |
| Males | 2r | 3r | 5r | 3.5r | | 4r | | - | | | | | |
|  | 1 | 85 | 4 | 3 | | 138 | |  |  |  |  |  |  |

**Supplementary Table 2: Characteristics of individuals included in the neuroimaging analyses**

|  | **DTI** | **Faces task** | **Resting state** | **N-back** |
| --- | --- | --- | --- | --- |
| Sample size (n) | 284 | 247 | 219 | 254 |
| Site (Mannheim/Berlin/Bonn) | 142 / 41/101 | 63 / 73 / 111 | 57 / 56 / 106 | 66 / 74 / 114 |
| *MAOA* genotype (H/L) | 200 / 84 | 175 / 72 | 155 / 64 | 179 / 75 |
| Sex (males/females) | 162 / 122 | 159 / 88 | 138 / 81 | 162 / 92 |
| Age (years), mean ± SD | 31.81 ± 10.23 | 34.38 ± 9.83 | 32.53 ± 9.69 | 34.30 ± 9.85 |
| Education (years), mean ± SD | 15.53 ± 2.49 | 15.42 ± 2.63 | 15.44 ± 2.53 | 15.39 ± 2.63 |

**Supplementary Table 3: Characteristics of individuals included in the sex-matched samples**

|  | **DTI** | **Faces task** | **Resting state** | **N-back** |
| --- | --- | --- | --- | --- |
| Sample size (n) | 244 | 176 | 162 | 184 |
| Site (Mannheim/Berlin/Bonn) | 122/37/85 | 46/56/74 | 42/42/78 | 49/52/83 |
| *MAOA* genotype (H/L) | 200/44 | 148/28 | 138/24 | 156/28 |
| Sex (males/females) | 122/122 | 88/88 | 81/81 | 92/92 |
| Age (years), mean ± SD | 31.62±10.29 | 34.53±9.77 | 32.30±9.71 | 34.17±9.81 |
| Education (years), mean ± SD | 15.47±2.46 | 15.24±2.52 | 15.33±2.41 | 15.21±2.51 |

MRI paradigms

The *emotional face matching task* ([Hariri et al., 2002](#_ENREF_4)) is an implicit emotion processing task consisting of an emotional face condition and a control condition. In the emotional face condition, participants were presented with trios of faces showing either angry or fearful expressions and were instructed to match the two facial displays belonging to the same individual. In the control condition, participants were presented with geometric shapes and were instructed to match the identical shapes. The task consisted of eight performance blocks of six trials or 30 s each, with alternating epochs of face- and form-matching conditions (task duration: 4.3 min or 130 whole-brain scans).

Working memory function was studied with an *n-back paradigm* ([Callicott et al., 2004](#_ENREF_1)). Briefly, a series of visual stimuli (numbers one to four) was displayed on a screen in a random order at set locations in a diamond-shaped box. Participants responded to each stimulus via a MRI compatible button box with four buttons arranged in the same configuration as the stimuli presented on the screen. In the 2-back working memory condition, participants were asked to encode the currently seen number, simultaneously recall the number seen two presentations previously, and press the button corresponding to the position of the number two presentations earlier. In the control condition (0-back), subjects were asked to press the button corresponding to the position of the current number presentation. The task was presented in eight blocks of 30 seconds each with alternating 0-back and 2-back conditions (task duration: 4.1 min or 124 whole-brain scans).

During the *resting state* scan ([Cao et al., 2014](#_ENREF_2)), participants were instructed to close their eyes, relax, and refrain from any particular mental activity (task duration: 5.0 min or 150 whole-brain scans). After each scan, investigators confirmed with the participant that they had not fallen asleep in the scanner.

**Supplemental Tables 4-6: Nodes and links of the MAOA-associated subnetworks**

For clarity of presentation, we projected the coordinates of nodes from significant links to the AAL atlas ([Tzourio-Mazoyer et al., 2002](#_ENREF_6)) and assigned the respective anatomical mask labels to the nodes. Coordinates are given in MNI space (x, y, z). Please note that the reported t-values of individual links are based on link-wise comparisons that are used to determine the initial network of the NBS analysis. Statistical significance of the NBS model is determined based on the number of connected links. Node labels mapping to the frontal cortex are bolded. Node labels corresponding to links connecting different nodes within the same major brain subdivision of the AAL atlas (isocoupled) are italicized. The top 10% of the most significant *MAOA*-associated links for each modality are highlighted in red.

Abbreviations: precentral gyrus (Precentral), superior frontal gyrus (Frontal_Sup), superior frontal gyrus, orbital part (Frontal_Sup_Orb), middle frontal gyrus (Frontal_Mid), middle frontal gyrus, orbital part (Frontal_Mid_Orb), inferior frontal gyrus, pars opercularis (Frontal_Inf_Oper), inferior frontal gyrus, pars triangularis (Frontal_Inf_Tri), inferior frontal gyrus, pars orbitalis (Frontal_Inf_Orb), Rolandic operculum (Rolandic_Oper), supplementary motor area (Supp_Motor_Area), olfactory cortex (Olfactory), medial frontal gyrus (Frontal_Sup_Medial), medial orbitofrontal cortex (Frontal_Med_Orb), gyrus rectus (Rectus), insula (Insula), anterior cingulate gyrus (Cingulum_Ant), midcingulate area (Cingulum_Mid), posterior cingulate gyrus (Cingulum_Post), hippocampus (Hippocampus), parahippocampal gyrus (ParaHippocampal), amydala (Amygdala), calcarine sulcus (Calcarine), cuneus (Cuneus), lingual gyrus (Lingual), superior occipital gyrus (Occipital_Sup), middle occipital gyrus (Occipital_Mid), inferior occipital gyrus (Occipital_Inf), fusiform gyrus (Fusiform), postcentral gyrus (Postcentral), superior parietal lobule (Parietal_Sup), inferior parietal lobule (Parietal_Inf), supramarginal gyrus (SupraMarginal), angular gyrus (Angular), precuneus (Precuneus), paracentral lobule (Paracentral_Lobule), caudate nucleus (Caudate), putamen (Putamen), globus pallidus (Pallidum), thalamus (Thalamus), transverse temporal gyrus (Heschl), superior temporal gyrus (Temporal_Sup), superior temporal pole (Temporal_Pole_Sup), middle temporal gyrus (Temporal_Mid), middle temporal pole (Temporal_Pole_Mid), inferior temporal gyrus (Temporal_Inf), crus I of cerebellar hemisphere (Cerebellum_Crus1), crus II of cerebellar hemisphere (Cerebellum_Crus2), lobule III of cerebellar hemisphere (Cerebellum_3), lobule IV, V of cerebellar hemisphere (Cerebellum_4_5), lobule VI of cerebellar hemisphere (Cerebellum_6), lobule VIIB of cerebellar hemisphere (Cerebellum_7b), lobule VIII of cerebellar hemisphere (Cerebellum_8), lobule IX of cerebellar hemisphere (Cerebellum_9), lobule X of cerebellar hemisphere (Cerebellum_10), Lobule I, II of vermis (Vermis_1_2), lobule III of vermis (Vermis_3), Lobule IV, V of vermis (Vermis_4_5), Lobule VI of vermis (Vermis_6), Lobule VII of vermis (Vermis_7), Lobule VIII of vermis (Vermis_8), Lobule IX of vermis (Vermis_9), Lobule X of vermis (Vermis_10). The succeeding “R.” and “L.” indicate the right and left hemisphere, respectively.

**fMRI emotion processing:**

| Brain node 1* | Brain node 2* | MNI coordinate 1# | MNI coordinate 2# | t-value | p-value |
| --- | --- | --- | --- | --- | --- |
| ***Precentral_R*** | ***Frontal_Inf_Oper_L*** | 40 -8 52 | -49 13 19 | 2.789244 | 0.002853 |
| ***Frontal_Inf_Oper_L*** | ***Frontal_Inf_Tri_R*** | -49 13 19 | 49 30 14 | 2.608844 | 0.004826 |
| ***Frontal_Inf_Oper_L*** | ***Frontal_Inf_Orb_R*** | -49 13 19 | 40 32 -12 | 2.670695 | 0.004042 |
| ***Frontal_Sup_L*** | ***Rolandic_Oper_L*** | -19 35 42 | -48 -8 14 | 2.62261 | 0.004641 |
| ***Frontal_Sup_Orb_L*** | ***Rolandic_Oper_L*** | -18 47 -13 | -48 -8 14 | 2.701509 | 0.003696 |
| ***Frontal_Mid_L*** | ***Rolandic_Oper_L*** | -34 33 35 | -48 -8 14 | 2.791957 | 0.00283 |
| ***Frontal_Mid_Orb_L*** | ***Rolandic_Oper_L*** | -32 50 -10 | -48 -8 14 | 3.338318 | 0.000488 |
| ***Frontal_Mid_Orb_R*** | ***Rolandic_Oper_L*** | 32 53 -11 | -48 -8 14 | 2.624582 | 0.004615 |
| ***Frontal_Inf_Orb_L*** | ***Rolandic_Oper_L*** | -37 31 -12 | -48 -8 14 | 2.701593 | 0.003695 |
| ***Frontal_Inf_Orb_R*** | ***Rolandic_Oper_L*** | 40 32 -12 | -48 -8 14 | 2.74621 | 0.003242 |
| ***Frontal_Inf_Orb_R*** | ***Rolandic_Oper_R*** | 40 32 -12 | 52 -6 15 | 2.664439 | 0.004116 |
| ***Frontal_Inf_Oper_L*** | ***Supp_Motor_Area_L*** | -49 13 19 | -6 5 61 | 3.065874 | 0.001209 |
| ***Frontal_Inf_Oper_L*** | ***Supp_Motor_Area_R*** | -49 13 19 | 8 0 62 | 2.931204 | 0.001851 |
| ***Rolandic_Oper_L*** | ***Frontal_Sup_Medial_L*** | -48 -8 14 | -6 49 31 | 3.007546 | 0.001456 |
| **Frontal_Inf_Oper_L** | Hippocampus_R | -49 13 19 | 28 -20 -10 | 2.894483 | 0.002073 |
| **Frontal_Inf_Tri_L** | Hippocampus_R | -47 30 14 | 28 -20 -10 | 2.672375 | 0.004023 |
| **Frontal_Inf_Oper_L** | ParaHippocampal_L | -49 13 19 | -22 -16 -21 | 2.831707 | 0.002511 |
| **Rolandic_Oper_L** | ParaHippocampal_L | -48 -8 14 | -22 -16 -21 | 2.784064 | 0.002897 |
| **Frontal_Inf_Oper_L** | ParaHippocampal_R | -49 13 19 | 24 -15 -20 | 3.443554 | 0.000338 |
| **Frontal_Inf_Tri_L** | ParaHippocampal_R | -47 30 14 | 24 -15 -20 | 3.072065 | 0.001185 |
| **Rolandic_Oper_L** | ParaHippocampal_R | -48 -8 14 | 24 -15 -20 | 2.808399 | 0.002693 |
| **Rolandic_Oper_R** | ParaHippocampal_R | 52 -6 15 | 24 -15 -20 | 2.887219 | 0.00212 |
| **Frontal_Inf_Oper_L** | Amygdala_L | -49 13 19 | -24 -1 -17 | 3.000939 | 0.001487 |
| **Frontal_Inf_Tri_L** | Amygdala_L | -47 30 14 | -24 -1 -17 | 2.953339 | 0.001727 |
| **Rolandic_Oper_L** | Amygdala_L | -48 -8 14 | -24 -1 -17 | 3.057512 | 0.001242 |
| **Rolandic_Oper_R** | Amygdala_L | 52 -6 15 | -24 -1 -17 | 3.310275 | 0.000537 |
| **Frontal_Inf_Oper_L** | Amygdala_R | -49 13 19 | 26 1 -18 | 3.012266 | 0.001435 |
| **Precentral_R** | Calcarine_L | 40 -8 52 | -8 -79 6 | 2.6922 | 0.003798 |
| **Frontal_Inf_Oper_L** | Calcarine_L | -49 13 19 | -8 -79 6 | 3.102684 | 0.001073 |
| **Rolandic_Oper_L** | Calcarine_L | -48 -8 14 | -8 -79 6 | 3.70804 | 0.00013 |
| **Rolandic_Oper_R** | Calcarine_L | 52 -6 15 | -8 -79 6 | 3.605841 | 0.000189 |
| **Precentral_R** | Calcarine_R | 40 -8 52 | 15 -73 9 | 2.792355 | 0.002826 |
| **Rolandic_Oper_L** | Calcarine_R | -48 -8 14 | 15 -73 9 | 2.89704 | 0.002057 |
| **Rolandic_Oper_R** | Calcarine_R | 52 -6 15 | 15 -73 9 | 2.86152 | 0.002293 |
| **Precentral_R** | Cuneus_L | 40 -8 52 | -7 -80 27 | 2.851949 | 0.002361 |
| **Rolandic_Oper_L** | Cuneus_L | -48 -8 14 | -7 -80 27 | 2.600447 | 0.004943 |
| **Precentral_R** | Cuneus_R | 40 -8 52 | 13 -79 28 | 2.66735 | 0.004082 |
| **Frontal_Inf_Oper_L** | Lingual_L | -49 13 19 | -16 -68 -5 | 3.18562 | 0.000818 |
| **Rolandic_Oper_L** | Lingual_L | -48 -8 14 | -16 -68 -5 | 3.604013 | 0.00019 |
| **Rolandic_Oper_R** | Lingual_L | 52 -6 15 | -16 -68 -5 | 3.342133 | 0.000482 |
| **Rolandic_Oper_L** | Lingual_R | -48 -8 14 | 15 -67 -4 | 3.419842 | 0.000368 |
| **Rolandic_Oper_R** | Lingual_R | 52 -6 15 | 15 -67 -4 | 2.971499 | 0.001632 |
| **Rolandic_Oper_L** | Occipital_Inf_L | -48 -8 14 | -37 -78 -8 | 2.639258 | 0.004425 |
| **Frontal_Inf_Oper_L** | Fusiform_L | -49 13 19 | -32 -40 -20 | 2.74551 | 0.003249 |
| **Rolandic_Oper_L** | Fusiform_L | -48 -8 14 | -32 -40 -20 | 2.76819 | 0.003037 |
| **Frontal_Inf_Oper_L** | Postcentral_R | -49 13 19 | 40 -25 53 | 2.665739 | 0.004101 |
| **Rolandic_Oper_L** | Postcentral_R | -48 -8 14 | 40 -25 53 | 2.701708 | 0.003694 |
| **Frontal_Mid_R** | Parietal_Inf_R | 37 33 34 | 45 -46 50 | 3.359739 | 0.000453 |
| **Frontal_Mid_Orb_R** | Parietal_Inf_R | 32 53 -11 | 45 -46 50 | 2.643273 | 0.004375 |
| **Frontal_Sup_L** | SupraMarginal_L | -19 35 42 | -57 -34 30 | 2.609817 | 0.004813 |
| **Frontal_Sup_Orb_L** | SupraMarginal_L | -18 47 -13 | -57 -34 30 | 2.993317 | 0.001523 |
| **Frontal_Sup_Orb_R** | SupraMarginal_L | 17 48 -14 | -57 -34 30 | 3.020688 | 0.001397 |
| **Frontal_Mid_L** | SupraMarginal_L | -34 33 35 | -57 -34 30 | 2.889888 | 0.002103 |
| **Frontal_Mid_R** | SupraMarginal_L | 37 33 34 | -57 -34 30 | 2.764285 | 0.003073 |
| **Frontal_Mid_Orb_L** | SupraMarginal_L | -32 50 -10 | -57 -34 30 | 3.339683 | 0.000486 |
| **Frontal_Mid_Orb_R** | SupraMarginal_L | 32 53 -11 | -57 -34 30 | 3.512509 | 0.000265 |
| **Frontal_Inf_Orb_L** | SupraMarginal_L | -37 31 -12 | -57 -34 30 | 2.903568 | 0.002016 |
| **Frontal_Inf_Orb_R** | SupraMarginal_L | 40 32 -12 | -57 -34 30 | 3.543148 | 0.000237 |
| **Frontal_Sup_Medial_L** | SupraMarginal_L | -6 49 31 | -57 -34 30 | 3.019961 | 0.0014 |
| **Frontal_Sup_Medial_R** | SupraMarginal_L | 8 51 30 | -57 -34 30 | 3.078757 | 0.00116 |
| **Frontal_Sup_R** | SupraMarginal_R | 20 31 44 | 57 -32 34 | 2.724862 | 0.003452 |
| **Frontal_Sup_Orb_R** | SupraMarginal_R | 17 48 -14 | 57 -32 34 | 2.976884 | 0.001604 |
| **Frontal_Mid_L** | SupraMarginal_R | -34 33 35 | 57 -32 34 | 2.975767 | 0.00161 |
| **Frontal_Mid_R** | SupraMarginal_R | 37 33 34 | 57 -32 34 | 3.438229 | 0.000345 |
| **Frontal_Mid_Orb_L** | SupraMarginal_R | -32 50 -10 | 57 -32 34 | 3.01103 | 0.00144 |
| **Frontal_Mid_Orb_R** | SupraMarginal_R | 32 53 -11 | 57 -32 34 | 3.328844 | 0.000504 |
| **Frontal_Inf_Orb_L** | SupraMarginal_R | -37 31 -12 | 57 -32 34 | 2.72396 | 0.003461 |
| **Frontal_Inf_Orb_R** | SupraMarginal_R | 40 32 -12 | 57 -32 34 | 3.519824 | 0.000258 |
| **Frontal_Sup_Medial_L** | SupraMarginal_R | -6 49 31 | 57 -32 34 | 2.598843 | 0.004965 |
| **Frontal_Sup_Medial_R** | SupraMarginal_R | 8 51 30 | 57 -32 34 | 2.765881 | 0.003058 |
| **Precentral_R** | Angular_R | 40 -8 52 | 45 -60 39 | 2.76286 | 0.003086 |
| **Frontal_Sup_Medial_R** | Angular_R | 8 51 30 | 45 -60 39 | 2.630354 | 0.00454 |
| **Frontal_Inf_Oper_L** | Putamen_L | -49 13 19 | -25 4 2 | 3.39865 | 0.000396 |
| **Rolandic_Oper_L** | Putamen_L | -48 -8 14 | -25 4 2 | 2.770999 | 0.003012 |
| **Rolandic_Oper_R** | Putamen_L | 52 -6 15 | -25 4 2 | 2.997316 | 0.001504 |
| **Frontal_Inf_Oper_L** | Putamen_R | -49 13 19 | 27 5 2 | 3.294054 | 0.000568 |
| **Rolandic_Oper_L** | Putamen_R | -48 -8 14 | 27 5 2 | 2.622979 | 0.004636 |
| **Rolandic_Oper_R** | Putamen_R | 52 -6 15 | 27 5 2 | 3.062953 | 0.00122 |
| **Frontal_Inf_Oper_L** | Pallidum_L | -49 13 19 | -19 0 0 | 3.496291 | 0.000281 |
| **Rolandic_Oper_L** | Pallidum_L | -48 -8 14 | -19 0 0 | 2.867384 | 0.002253 |
| **Rolandic_Oper_R** | Pallidum_L | 52 -6 15 | -19 0 0 | 3.103917 | 0.001069 |
| **Frontal_Inf_Oper_L** | Pallidum_R | -49 13 19 | 20 0 0 | 3.355868 | 0.00046 |
| **Rolandic_Oper_L** | Pallidum_R | -48 -8 14 | 20 0 0 | 2.966931 | 0.001655 |
| **Rolandic_Oper_R** | Pallidum_R | 52 -6 15 | 20 0 0 | 3.251025 | 0.000657 |
| **Precentral_R** | Heschl_L | 40 -8 52 | -43 -19 10 | 2.834491 | 0.00249 |
| **Frontal_Sup_Orb_L** | Heschl_L | -18 47 -13 | -43 -19 10 | 2.714338 | 0.00356 |
| **Precentral_L** | Heschl_R | -40 -6 51 | 45 -17 10 | 2.893906 | 0.002077 |
| **Frontal_Inf_Oper_L** | Heschl_R | -49 13 19 | 45 -17 10 | 3.165487 | 0.000874 |
| **Frontal_Inf_Tri_L** | Heschl_R | -47 30 14 | 45 -17 10 | 2.928633 | 0.001865 |
| **Frontal_Sup_Medial_L** | Heschl_R | -6 49 31 | 45 -17 10 | 2.619154 | 0.004687 |
| **Precentral_L** | Temporal_Sup_L | -40 -6 51 | -54 -21 7 | 2.762779 | 0.003087 |
| **Precentral_R** | Temporal_Sup_L | 40 -8 52 | -54 -21 7 | 3.175672 | 0.000845 |
| **Frontal_Sup_L** | Temporal_Sup_L | -19 35 42 | -54 -21 7 | 3.009291 | 0.001448 |
| **Frontal_Sup_Orb_L** | Temporal_Sup_L | -18 47 -13 | -54 -21 7 | 3.20227 | 0.000774 |
| **Frontal_Mid_L** | Temporal_Sup_L | -34 33 35 | -54 -21 7 | 3.175854 | 0.000844 |
| **Frontal_Mid_Orb_L** | Temporal_Sup_L | -32 50 -10 | -54 -21 7 | 2.650256 | 0.004288 |
| **Frontal_Inf_Oper_L** | Temporal_Sup_L | -49 13 19 | -54 -21 7 | 2.940814 | 0.001796 |
| **Frontal_Inf_Orb_L** | Temporal_Sup_L | -37 31 -12 | -54 -21 7 | 2.63616 | 0.004465 |
| **Frontal_Sup_Medial_L** | Temporal_Sup_L | -6 49 31 | -54 -21 7 | 3.596051 | 0.000196 |
| **Frontal_Sup_Medial_R** | Temporal_Sup_L | 8 51 30 | -54 -21 7 | 3.201194 | 0.000776 |
| **Frontal_Inf_Oper_L** | Temporal_Sup_R | -49 13 19 | 57 -22 7 | 3.118342 | 0.00102 |
| **Rolandic_Oper_L** | Temporal_Pole_Sup_L | -48 -8 14 | -41 15 -20 | 2.627002 | 0.004583 |
| **Frontal_Inf_Oper_L** | Temporal_Pole_Sup_R | -49 13 19 | 47 15 -17 | 3.370703 | 0.000437 |
| **Frontal_Inf_Tri_L** | Temporal_Pole_Sup_R | -47 30 14 | 47 15 -17 | 3.040713 | 0.00131 |
| **Rolandic_Oper_L** | Temporal_Pole_Sup_R | -48 -8 14 | 47 15 -17 | 2.835791 | 0.00248 |
| **Supp_Motor_Area_L** | Temporal_Pole_Sup_R | -6 5 61 | 47 15 -17 | 2.718938 | 0.003513 |
| **Precentral_R** | Temporal_Mid_L | 40 -8 52 | -57 -34 -2 | 2.808383 | 0.002694 |
| **Frontal_Inf_Oper_L** | Temporal_Mid_L | -49 13 19 | -57 -34 -2 | 3.396092 | 0.0004 |
| **Rolandic_Oper_L** | Temporal_Mid_L | -48 -8 14 | -57 -34 -2 | 2.877629 | 0.002183 |
| **Supp_Motor_Area_R** | Temporal_Mid_L | 8 0 62 | -57 -34 -2 | 2.70887 | 0.003618 |
| **Frontal_Inf_Oper_L** | Temporal_Mid_R | -49 13 19 | 56 -37 -1 | 2.838276 | 0.002461 |
| **Rolandic_Oper_L** | Temporal_Mid_R | -48 -8 14 | 56 -37 -1 | 2.794799 | 0.002806 |
| **Frontal_Inf_Oper_L** | Cerebellum_3_L | -49 13 19 | -9 -37 -19 | 3.030009 | 0.001356 |
| **Frontal_Inf_Oper_L** | Cerebellum_4_5_L | -49 13 19 | -15 -43 -17 | 3.143643 | 0.000939 |
| **Rolandic_Oper_L** | Cerebellum_4_5_L | -48 -8 14 | -15 -43 -17 | 3.116455 | 0.001026 |
| **Frontal_Inf_Oper_L** | Cerebellum_6_L | -49 13 19 | -23 -59 -22 | 2.912824 | 0.001959 |
| **Rolandic_Oper_L** | Cerebellum_6_L | -48 -8 14 | -23 -59 -22 | 3.276311 | 0.000603 |
| **Rolandic_Oper_L** | Cerebellum_6_R | -48 -8 14 | 25 -58 -24 | 2.729007 | 0.00341 |
| **Rolandic_Oper_R** | Cerebellum_6_R | 52 -6 15 | 25 -58 -24 | 2.626579 | 0.004589 |
| **Rolandic_Oper_L** | Cerebellum_7b_L | -48 -8 14 | -33 -60 -43 | 2.975994 | 0.001609 |
| **Frontal_Inf_Oper_L** | Cerebellum_8_L | -49 13 19 | -26 -55 -48 | 2.655705 | 0.004221 |
| **Rolandic_Oper_L** | Cerebellum_8_L | -48 -8 14 | -26 -55 -48 | 3.150936 | 0.000917 |
| **Frontal_Sup_L** | Cerebellum_9_L | -19 35 42 | -11 -49 -46 | 2.765881 | 0.003058 |
| **Frontal_Sup_R** | Cerebellum_9_L | 20 31 44 | -11 -49 -46 | 2.808698 | 0.002691 |
| **Rolandic_Oper_L** | Cerebellum_9_L | -48 -8 14 | -11 -49 -46 | 2.813476 | 0.002653 |
| **Frontal_Sup_Medial_R** | Cerebellum_9_L | 8 51 30 | -11 -49 -46 | 2.864813 | 0.00227 |
| **Frontal_Inf_Oper_L** | Vermis_1_2 | -49 13 19 | 1 -39 -20 | 2.779338 | 0.002938 |
| **Rolandic_Oper_L** | Vermis_7 | -48 -8 14 | 1 -72 -25 | 2.811836 | 0.002666 |
| **Frontal_Inf_Oper_L** | Vermis_10 | -49 13 19 | 0 -46 -32 | 2.782079 | 0.002914 |
| *Insula_L* | *Amygdala_L* | -36 7 3 | -24 -1 -17 | 2.888621 | 0.002111 |
| *Insula_R* | *Amygdala_L* | 38 6 2 | -24 -1 -17 | 3.218185 | 0.000734 |
| Insula_L | Postcentral_R | -36 7 3 | 40 -25 53 | 2.738212 | 0.003319 |
| *ParaHippocampal_L* | *Heschl_L* | -22 -16 -21 | -43 -19 10 | 2.816006 | 0.002632 |
| *Amygdala_L* | *Heschl_L* | -24 -1 -17 | -43 -19 10 | 2.868592 | 0.002244 |
| *Fusiform_L* | *Heschl_L* | -32 -40 -20 | -43 -19 10 | 2.834629 | 0.002489 |
| *Insula_L* | *Heschl_R* | -36 7 3 | 45 -17 10 | 2.623471 | 0.00463 |
| *Amygdala_L* | *Heschl_R* | -24 -1 -17 | 45 -17 10 | 3.171374 | 0.000857 |
| *Insula_L* | *Temporal_Sup_R* | -36 7 3 | 57 -22 7 | 2.709538 | 0.003611 |
| *Temporal_Sup_L* | *Temporal_Sup_R* | -54 -21 7 | 57 -22 7 | 2.856756 | 0.002327 |
| *Heschl_R* | *Temporal_Pole_Sup_L* | 45 -17 10 | -41 15 -20 | 2.717225 | 0.00353 |
| *Temporal_Sup_L* | *Temporal_Mid_L* | -54 -21 7 | -57 -34 -2 | 2.786839 | 0.002873 |
| *Temporal_Sup_L* | *Temporal_Mid_R* | -54 -21 7 | 56 -37 -1 | 2.597953 | 0.004978 |
| *Fusiform_L* | *Temporal_Pole_Mid_L* | -32 -40 -20 | -37 15 -34 | 2.804776 | 0.002723 |
| *Fusiform_R* | *Temporal_Pole_Mid_L* | 33 -39 -20 | -37 15 -34 | 2.740031 | 0.003301 |
| *ParaHippocampal_R* | *Temporal_Pole_Mid_R* | 24 -15 -20 | 43 15 -32 | 2.968823 | 0.001646 |
| *Fusiform_R* | *Temporal_Pole_Mid_R* | 33 -39 -20 | 43 15 -32 | 2.63286 | 0.004507 |
| *Heschl_L* | *Temporal_Inf_L* | -43 -19 10 | -51 -28 -23 | 2.683405 | 0.003896 |
| Amygdala_R | Cerebellum_4_5_L | 26 1 -18 | -15 -43 -17 | 2.799043 | 0.00277 |
| Heschl_L | Cerebellum_4_5_L | -43 -19 10 | -15 -43 -17 | 2.682549 | 0.003906 |
| Heschl_L | Cerebellum_6_L | -43 -19 10 | -23 -59 -22 | 3.194575 | 0.000794 |
| Temporal_Sup_L | Cerebellum_6_L | -54 -21 7 | -23 -59 -22 | 3.083956 | 0.00114 |
| Heschl_L | Cerebellum_6_R | -43 -19 10 | 25 -58 -24 | 2.625821 | 0.004599 |
| Heschl_L | Cerebellum_7b_L | -43 -19 10 | -33 -60 -43 | 3.011118 | 0.00144 |
| ParaHippocampal_R | Cerebellum_7b_R | 24 -15 -20 | 35 -64 -47 | 2.609186 | 0.004822 |
| Heschl_L | Cerebellum_8_L | -43 -19 10 | -26 -55 -48 | 3.123467 | 0.001003 |
| Heschl_L | Cerebellum_8_R | -43 -19 10 | 25 -56 -49 | 2.647387 | 0.004323 |
| ParaHippocampal_L | Cerebellum_9_L | -22 -16 -21 | -11 -49 -46 | 2.822023 | 0.002585 |
| ParaHippocampal_R | Cerebellum_9_L | 24 -15 -20 | -11 -49 -46 | 2.77425 | 0.002983 |
| Fusiform_L | Cerebellum_9_L | -32 -40 -20 | -11 -49 -46 | 2.680481 | 0.00393 |
| Fusiform_R | Cerebellum_9_L | 33 -39 -20 | -11 -49 -46 | 3.032645 | 0.001345 |
| Heschl_L | Cerebellum_9_L | -43 -19 10 | -11 -49 -46 | 2.741878 | 0.003284 |
| Temporal_Inf_R | Cerebellum_9_L | 53 -31 -22 | -11 -49 -46 | 2.842775 | 0.002428 |
| ParaHippocampal_R | Cerebellum_9_R | 24 -15 -20 | 9 -49 -46 | 2.622846 | 0.004638 |
| Fusiform_L | Cerebellum_9_R | -32 -40 -20 | 9 -49 -46 | 2.82647 | 0.002551 |
| Fusiform_R | Cerebellum_9_R | 33 -39 -20 | 9 -49 -46 | 3.05025 | 0.001271 |
| Heschl_L | Cerebellum_9_R | -43 -19 10 | 9 -49 -46 | 2.64633 | 0.004336 |
| Temporal_Inf_R | Cerebellum_9_R | 53 -31 -22 | 9 -49 -46 | 2.846208 | 0.002403 |
| Heschl_L | Vermis_8 | -43 -19 10 | 1 -64 -34 | 2.648404 | 0.004311 |
| Fusiform_R | Vermis_10 | 33 -39 -20 | 0 -46 -32 | 2.727145 | 0.003429 |
| Temporal_Pole_Mid_R | Vermis_10 | 43 15 -32 | 0 -46 -32 | 2.80612 | 0.002712 |
| *Parietal_Inf_L* | *SupraMarginal_R* | -44 -46 47 | 57 -32 34 | 2.653946 | 0.004243 |
| *Parietal_Inf_R* | *SupraMarginal_R* | 45 -46 50 | 57 -32 34 | 3.596016 | 0.000196 |
| *Postcentral_R* | *Angular_R* | 40 -25 53 | 45 -60 39 | 2.767595 | 0.003043 |
| Parietal_Inf_L | Putamen_L | -44 -46 47 | -25 4 2 | 2.667323 | 0.004082 |
| SupraMarginal_L | Putamen_L | -57 -34 30 | -25 4 2 | 2.808417 | 0.002693 |
| SupraMarginal_L | Putamen_R | -57 -34 30 | 27 5 2 | 2.725411 | 0.003447 |
| SupraMarginal_R | Putamen_R | 57 -32 34 | 27 5 2 | 2.639713 | 0.00442 |
| Postcentral_L | Pallidum_L | -43 -23 49 | -19 0 0 | 2.646974 | 0.004328 |
| Parietal_Sup_L | Pallidum_L | -24 -60 59 | -19 0 0 | 2.818748 | 0.002611 |
| Parietal_Inf_L | Pallidum_L | -44 -46 47 | -19 0 0 | 2.915913 | 0.00194 |
| Parietal_Inf_R | Pallidum_L | 45 -46 50 | -19 0 0 | 2.698369 | 0.00373 |
| SupraMarginal_L | Pallidum_L | -57 -34 30 | -19 0 0 | 3.064196 | 0.001215 |
| SupraMarginal_R | Pallidum_L | 57 -32 34 | -19 0 0 | 3.001252 | 0.001486 |
| Parietal_Inf_L | Pallidum_R | -44 -46 47 | 20 0 0 | 2.610988 | 0.004797 |
| SupraMarginal_L | Pallidum_R | -57 -34 30 | 20 0 0 | 2.678445 | 0.003953 |
| SupraMarginal_R | Pallidum_R | 57 -32 34 | 20 0 0 | 2.80597 | 0.002713 |
| Postcentral_R | Heschl_L | 40 -25 53 | -43 -19 10 | 3.232439 | 0.000699 |
| Postcentral_R | Heschl_R | 40 -25 53 | 45 -17 10 | 2.87488 | 0.002202 |
| Postcentral_R | Temporal_Sup_L | 40 -25 53 | -54 -21 7 | 3.511396 | 0.000266 |
| Parietal_Sup_L | Temporal_Sup_L | -24 -60 59 | -54 -21 7 | 2.600494 | 0.004942 |
| Parietal_Inf_R | Temporal_Sup_L | 45 -46 50 | -54 -21 7 | 2.850556 | 0.002371 |
| Postcentral_R | Temporal_Sup_R | 40 -25 53 | 57 -22 7 | 2.892967 | 0.002083 |
| SupraMarginal_L | Temporal_Sup_R | -57 -34 30 | 57 -22 7 | 2.623751 | 0.004626 |
| SupraMarginal_L | Temporal_Mid_L | -57 -34 30 | -57 -34 -2 | 2.689875 | 0.003824 |
| SupraMarginal_R | Temporal_Mid_L | 57 -32 34 | -57 -34 -2 | 2.656672 | 0.004209 |
| SupraMarginal_R | Temporal_Mid_R | 57 -32 34 | 56 -37 -1 | 2.672157 | 0.004025 |
| Postcentral_R | Cerebellum_4_5_L | 40 -25 53 | -15 -43 -17 | 2.63115 | 0.004529 |
| Parietal_Inf_R | Cerebellum_6_L | 45 -46 50 | -23 -59 -22 | 3.190445 | 0.000805 |
| SupraMarginal_L | Cerebellum_6_L | -57 -34 30 | -23 -59 -22 | 2.624361 | 0.004618 |
| SupraMarginal_R | Cerebellum_6_L | 57 -32 34 | -23 -59 -22 | 2.723474 | 0.003466 |
| Angular_R | Cerebellum_6_L | 45 -60 39 | -23 -59 -22 | 3.02152 | 0.001393 |
| SupraMarginal_L | Cerebellum_9_L | -57 -34 30 | -11 -49 -46 | 2.675232 | 0.00399 |
| Angular_R | Cerebellum_9_L | 45 -60 39 | -11 -49 -46 | 2.724065 | 0.00346 |
| Angular_R | Cerebellum_9_R | 45 -60 39 | 9 -49 -46 | 2.696723 | 0.003748 |
| Lingual_L | Postcentral_L | -16 -68 -5 | -43 -23 49 | 2.608688 | 0.004829 |
| Calcarine_L | Postcentral_R | -8 -79 6 | 40 -25 53 | 2.685396 | 0.003874 |
| Calcarine_R | Postcentral_R | 15 -73 9 | 40 -25 53 | 2.958247 | 0.001701 |
| Cuneus_L | Postcentral_R | -7 -80 27 | 40 -25 53 | 2.95643 | 0.001711 |
| Lingual_L | Postcentral_R | -16 -68 -5 | 40 -25 53 | 2.655342 | 0.004226 |
| Calcarine_L | SupraMarginal_L | -8 -79 6 | -57 -34 30 | 2.869188 | 0.00224 |
| Cuneus_L | SupraMarginal_L | -7 -80 27 | -57 -34 30 | 2.607824 | 0.00484 |
| Calcarine_L | SupraMarginal_R | -8 -79 6 | 57 -32 34 | 3.251247 | 0.000657 |
| Calcarine_R | SupraMarginal_R | 15 -73 9 | 57 -32 34 | 2.715451 | 0.003549 |
| Lingual_L | SupraMarginal_R | -16 -68 -5 | 57 -32 34 | 2.750953 | 0.003197 |
| Occipital_Sup_L | SupraMarginal_R | -18 -84 28 | 57 -32 34 | 2.662391 | 0.004141 |
| Calcarine_L | Heschl_L | -8 -79 6 | -43 -19 10 | 2.964357 | 0.001669 |
| Lingual_L | Heschl_L | -16 -68 -5 | -43 -19 10 | 2.718108 | 0.003521 |
| Calcarine_L | Heschl_R | -8 -79 6 | 45 -17 10 | 3.034207 | 0.001338 |
| Lingual_L | Heschl_R | -16 -68 -5 | 45 -17 10 | 3.158041 | 0.000896 |
| Lingual_R | Heschl_R | 15 -67 -4 | 45 -17 10 | 2.725905 | 0.003442 |
| Calcarine_L | Temporal_Sup_L | -8 -79 6 | -54 -21 7 | 3.137581 | 0.000958 |
| Lingual_L | Temporal_Sup_L | -16 -68 -5 | -54 -21 7 | 2.944979 | 0.001773 |
| Calcarine_L | Temporal_Sup_R | -8 -79 6 | 57 -22 7 | 2.689129 | 0.003832 |
| Cuneus_R | Temporal_Mid_L | 13 -79 28 | -57 -34 -2 | 2.604252 | 0.00489 |
| Occipital_Sup_L | Temporal_Mid_L | -18 -84 28 | -57 -34 -2 | 2.643959 | 0.004366 |
| Cingulum_Post_L | Amygdala_R | -6 -43 25 | 26 1 -18 | 2.650216 | 0.004288 |
| Cingulum_Post_R | Amygdala_R | 6 -42 22 | 26 1 -18 | 2.843841 | 0.00242 |
| Cingulum_Mid_L | Calcarine_L | -6 -15 42 | -8 -79 6 | 2.699047 | 0.003723 |
| Cingulum_Mid_R | Calcarine_L | 7 -9 40 | -8 -79 6 | 2.843981 | 0.002419 |
| Cingulum_Mid_L | Calcarine_R | -6 -15 42 | 15 -73 9 | 2.678941 | 0.003947 |
| Cingulum_Mid_L | Cuneus_L | -6 -15 42 | -7 -80 27 | 2.604551 | 0.004886 |
| Pallidum_L | Heschl_L | -19 0 0 | -43 -19 10 | 2.65299 | 0.004254 |
| *Putamen_L* | *Cerebellum_9_L* | -25 4 2 | -11 -49 -46 | 2.871398 | 0.002225 |
| *Cerebellum_4_5_L* | *Cerebellum_9_L* | -15 -43 -17 | -11 -49 -46 | 2.708632 | 0.00362 |
| *Cerebellum_6_R* | *Cerebellum_9_L* | 25 -58 -24 | -11 -49 -46 | 2.609321 | 0.00482 |
| *Putamen_L* | *Cerebellum_9_R* | -25 4 2 | 9 -49 -46 | 2.617878 | 0.004704 |
| *Cerebellum_4_5_L* | *Cerebellum_9_R* | -15 -43 -17 | 9 -49 -46 | 3.465129 | 0.000314 |
| *Cerebellum_4_5_R* | *Cerebellum_9_R* | 17 -43 -18 | 9 -49 -46 | 2.898954 | 0.002045 |
| *Cerebellum_6_R* | *Cerebellum_9_R* | 25 -58 -24 | 9 -49 -46 | 2.705926 | 0.003649 |
| *Cerebellum_9_L* | *Vermis_7* | -11 -49 -46 | 1 -72 -25 | 3.040696 | 0.001311 |
| *Cerebellum_9_R* | *Vermis_7* | 9 -49 -46 | 1 -72 -25 | 2.773311 | 0.002991 |
| *Cerebellum_9_L* | *Vermis_8* | -11 -49 -46 | 1 -64 -34 | 3.233686 | 0.000696 |
| *Cerebellum_9_R* | *Vermis_8* | 9 -49 -46 | 1 -64 -34 | 2.921687 | 0.001906 |
| *Vermis_7* | *Vermis_9* | 1 -72 -25 | 1 -55 -35 | 2.704707 | 0.003662 |
| *Vermis_8* | *Vermis_9* | 1 -64 -34 | 1 -55 -35 | 3.296187 | 0.000564 |
| *Cerebellum_3_L* | *Vermis_10* | -9 -37 -19 | 0 -46 -32 | 3.103545 | 0.00107 |
| *Cerebellum_4_5_L* | *Vermis_10* | -15 -43 -17 | 0 -46 -32 | 3.108277 | 0.001054 |
| *Vermis_1_2* | *Vermis_10* | 1 -39 -20 | 0 -46 -32 | 2.737448 | 0.003327 |

**fMRI resting state:**

| Brain node 1* | Brain node 2* | MNI coordinate 1# | MNI coordinate 2# | t-value | p-value |
| --- | --- | --- | --- | --- | --- |
| ***Precentral_L*** | ***Precentral_R*** | -40 -6 51 | 40 -8 52 | 3.310026 | 0.000548 |
| ***Frontal_Sup_L*** | ***Frontal_Sup_Orb_L*** | -19 35 42 | -18 47 -13 | 2.957296 | 0.001727 |
| ***Frontal_Sup_L*** | ***Frontal_Sup_Orb_R*** | -19 35 42 | 17 48 -14 | 3.42589 | 0.000368 |
| ***Frontal_Sup_Orb_R*** | ***Frontal_Mid_L*** | 17 48 -14 | -34 33 35 | 3.102135 | 0.001091 |
| ***Frontal_Mid_Orb_R*** | ***Frontal_Inf_Oper_R*** | 32 53 -11 | 49 15 21 | 2.661537 | 0.004186 |
| ***Precentral_L*** | ***Rolandic_Oper_L*** | -40 -6 51 | -48 -8 14 | 2.770618 | 0.003045 |
| ***Precentral_L*** | ***Rolandic_Oper_R*** | -40 -6 51 | 52 -6 15 | 3.065516 | 0.001227 |
| ***Rolandic_Oper_L*** | ***Rolandic_Oper_R*** | -48 -8 14 | 52 -6 15 | 2.715473 | 0.003581 |
| ***Frontal_Sup_L*** | ***Olfactory_L*** | -19 35 42 | -9 15 -12 | 3.24721 | 0.000677 |
| ***Frontal_Sup_Orb_L*** | ***Frontal_Sup_Medial_L*** | -18 47 -13 | -6 49 31 | 2.764868 | 0.003097 |
| ***Frontal_Sup_Orb_R*** | ***Frontal_Sup_Medial_L*** | 17 48 -14 | -6 49 31 | 3.392131 | 0.000413 |
| ***Olfactory_L*** | ***Frontal_Sup_Medial_L*** | -9 15 -12 | -6 49 31 | 2.678632 | 0.003985 |
| ***Olfactory_R*** | ***Frontal_Sup_Medial_L*** | 8 16 -11 | -6 49 31 | 2.700088 | 0.003745 |
| ***Olfactory_L*** | ***Rectus_L*** | -9 15 -12 | -6 37 -18 | 2.647651 | 0.004356 |
| ***Olfactory_R*** | ***Rectus_L*** | 8 16 -11 | -6 37 -18 | 2.805089 | 0.002748 |
| ***Rectus_L*** | ***Rectus_R*** | -6 37 -18 | 7 36 -18 | 3.076873 | 0.001183 |
| **Frontal_Mid_Orb_L** | Insula_L | -32 50 -10 | -36 7 3 | 2.648647 | 0.004343 |
| **Frontal_Inf_Oper_L** | Insula_L | -49 13 19 | -36 7 3 | 2.747888 | 0.003256 |
| **Frontal_Mid_Orb_L** | Insula_R | -32 50 -10 | 38 6 2 | 2.810025 | 0.002708 |
| **Frontal_Inf_Orb_L** | Insula_R | -37 31 -12 | 38 6 2 | 2.669163 | 0.004095 |
| **Frontal_Med_Orb_L** | ParaHippocampal_L | -6 54 -7 | -22 -16 -21 | 3.052396 | 0.00128 |
| **Rectus_L** | ParaHippocampal_L | -6 37 -18 | -22 -16 -21 | 2.707983 | 0.00366 |
| **Precentral_R** | Amygdala_L | 40 -8 52 | -24 -1 -17 | 2.691636 | 0.003838 |
| **Rolandic_Oper_L** | Amygdala_L | -48 -8 14 | -24 -1 -17 | 3.030543 | 0.001372 |
| **Rolandic_Oper_R** | Amygdala_L | 52 -6 15 | -24 -1 -17 | 3.192868 | 0.000811 |
| **Frontal_Med_Orb_L** | Amygdala_L | -6 54 -7 | -24 -1 -17 | 3.118694 | 0.001034 |
| **Rectus_L** | Amygdala_L | -6 37 -18 | -24 -1 -17 | 2.677342 | 0.003999 |
| **Frontal_Med_Orb_L** | Fusiform_L | -6 54 -7 | -32 -40 -20 | 2.918419 | 0.001948 |
| **Precentral_L** | Heschl_L | -40 -6 51 | -43 -19 10 | 2.623463 | 0.004667 |
| **Frontal_Inf_Oper_L** | Heschl_L | -49 13 19 | -43 -19 10 | 2.880777 | 0.002186 |
| **Frontal_Inf_Tri_L** | Heschl_L | -47 30 14 | -43 -19 10 | 2.606768 | 0.004893 |
| **Rolandic_Oper_L** | Heschl_L | -48 -8 14 | -43 -19 10 | 3.252335 | 0.000665 |
| **Frontal_Med_Orb_L** | Heschl_L | -6 54 -7 | -43 -19 10 | 2.603532 | 0.004938 |
| **Precentral_L** | Heschl_R | -40 -6 51 | 45 -17 10 | 3.283419 | 0.000599 |
| **Precentral_R** | Heschl_R | 40 -8 52 | 45 -17 10 | 3.004683 | 0.001489 |
| **Frontal_Inf_Oper_L** | Heschl_R | -49 13 19 | 45 -17 10 | 2.831648 | 0.002537 |
| **Rolandic_Oper_L** | Heschl_R | -48 -8 14 | 45 -17 10 | 2.845439 | 0.002434 |
| **Frontal_Med_Orb_L** | Heschl_R | -6 54 -7 | 45 -17 10 | 2.679178 | 0.003978 |
| **Frontal_Inf_Oper_L** | Temporal_Sup_R | -49 13 19 | 57 -22 7 | 2.767341 | 0.003074 |
| **Frontal_Mid_Orb_R** | Temporal_Pole_Sup_R | 32 53 -11 | 47 15 -17 | 2.887549 | 0.002141 |
| **Frontal_Sup_R** | Temporal_Mid_L | 20 31 44 | -57 -34 -2 | 2.837863 | 0.00249 |
| **Frontal_Sup_Orb_R** | Temporal_Mid_L | 17 48 -14 | -57 -34 -2 | 3.139313 | 0.000967 |
| **Frontal_Mid_R** | Temporal_Mid_L | 37 33 34 | -57 -34 -2 | 3.404252 | 0.000396 |
| **Frontal_Inf_Tri_R** | Temporal_Mid_L | 49 30 14 | -57 -34 -2 | 2.737197 | 0.00336 |
| **Frontal_Sup_Orb_R** | Temporal_Inf_L | 17 48 -14 | -51 -28 -23 | 2.908232 | 0.00201 |
| **Supp_Motor_Area_L** | Temporal_Inf_L | -6 5 61 | -51 -28 -23 | 2.803213 | 0.002763 |
| **Supp_Motor_Area_R** | Temporal_Inf_L | 8 0 62 | -51 -28 -23 | 2.873163 | 0.002238 |
| **Precentral_L** | Postcentral_L | -40 -6 51 | -43 -23 49 | 2.910698 | 0.001995 |
| **Precentral_L** | Postcentral_R | -40 -6 51 | 40 -25 53 | 2.776823 | 0.002989 |
| **Frontal_Mid_Orb_L** | SupraMarginal_L | -32 50 -10 | -57 -34 30 | 3.124828 | 0.001013 |
| **Frontal_Mid_Orb_R** | SupraMarginal_L | 32 53 -11 | -57 -34 30 | 2.604744 | 0.004921 |
| **Frontal_Inf_Oper_L** | SupraMarginal_L | -49 13 19 | -57 -34 30 | 3.806711 | 0.000092 |
| **Frontal_Inf_Tri_L** | SupraMarginal_L | -47 30 14 | -57 -34 30 | 3.333842 | 0.000505 |
| **Frontal_Mid_Orb_L** | SupraMarginal_R | -32 50 -10 | 57 -32 34 | 2.669335 | 0.004093 |
| **Frontal_Mid_Orb_R** | SupraMarginal_R | 32 53 -11 | 57 -32 34 | 2.843103 | 0.002451 |
| **Frontal_Inf_Tri_L** | SupraMarginal_R | -47 30 14 | 57 -32 34 | 2.878164 | 0.002204 |
| **Frontal_Sup_Orb_R** | Angular_L | 17 48 -14 | -45 -61 36 | 2.635808 | 0.004506 |
| **Frontal_Inf_Tri_R** | Angular_L | 49 30 14 | -45 -61 36 | 2.603538 | 0.004938 |
| **Supp_Motor_Area_R** | Angular_L | 8 0 62 | -45 -61 36 | 2.64333 | 0.00441 |
| **Frontal_Med_Orb_R** | Angular_R | 7 52 -7 | 45 -60 39 | 2.664605 | 0.004149 |
| **Frontal_Sup_Orb_R** | Calcarine_L | 17 48 -14 | -8 -79 6 | 3.02667 | 0.001389 |
| **Frontal_Med_Orb_L** | Calcarine_L | -6 54 -7 | -8 -79 6 | 2.840392 | 0.002471 |
| **Rectus_L** | Calcarine_L | -6 37 -18 | -8 -79 6 | 3.112357 | 0.001055 |
| **Rectus_R** | Calcarine_L | 7 36 -18 | -8 -79 6 | 3.021908 | 0.00141 |
| **Frontal_Med_Orb_L** | Calcarine_R | -6 54 -7 | 15 -73 9 | 3.084391 | 0.001155 |
| **Rectus_L** | Calcarine_R | -6 37 -18 | 15 -73 9 | 2.942496 | 0.001808 |
| **Rectus_R** | Calcarine_R | 7 36 -18 | 15 -73 9 | 2.780431 | 0.002957 |
| **Frontal_Med_Orb_L** | Lingual_L | -6 54 -7 | -16 -68 -5 | 2.760344 | 0.003139 |
| **Frontal_Med_Orb_L** | Lingual_R | -6 54 -7 | 15 -67 -4 | 2.670781 | 0.004076 |
| **Rectus_L** | Occipital_Sup_L | -6 37 -18 | -18 -84 28 | 2.651426 | 0.004309 |
| **Frontal_Med_Orb_L** | Occipital_Sup_R | -6 54 -7 | 23 -81 31 | 2.703143 | 0.003712 |
| **Frontal_Inf_Oper_L** | Occipital_Mid_L | -49 13 19 | -33 -81 16 | 2.809851 | 0.002709 |
| **Frontal_Med_Orb_L** | Occipital_Mid_L | -6 54 -7 | -33 -81 16 | 2.658799 | 0.004219 |
| **Frontal_Mid_L** | Occipital_Mid_R | -34 33 35 | 36 -80 19 | 2.727978 | 0.003452 |
| **Frontal_Inf_Oper_L** | Occipital_Mid_R | -49 13 19 | 36 -80 19 | 2.623193 | 0.00467 |
| **Frontal_Med_Orb_L** | Occipital_Mid_R | -6 54 -7 | 36 -80 19 | 2.934207 | 0.001855 |
| **Frontal_Inf_Oper_L** | Occipital_Inf_L | -49 13 19 | -37 -78 -8 | 3.082428 | 0.001162 |
| **Frontal_Med_Orb_L** | Occipital_Inf_R | -6 54 -7 | 37 -82 -8 | 2.662238 | 0.004177 |
| **Rectus_L** | Occipital_Inf_R | -6 37 -18 | 37 -82 -8 | 2.631176 | 0.004565 |
| **Frontal_Sup_Orb_L** | Putamen_L | -18 47 -13 | -25 4 2 | 3.169034 | 0.000877 |
| **Frontal_Sup_Orb_R** | Putamen_L | 17 48 -14 | -25 4 2 | 3.408039 | 0.000391 |
| **Frontal_Mid_L** | Putamen_L | -34 33 35 | -25 4 2 | 2.69068 | 0.003848 |
| **Frontal_Mid_R** | Putamen_L | 37 33 34 | -25 4 2 | 2.730799 | 0.003424 |
| **Frontal_Mid_Orb_L** | Putamen_L | -32 50 -10 | -25 4 2 | 2.937998 | 0.001834 |
| **Frontal_Mid_Orb_R** | Putamen_L | 32 53 -11 | -25 4 2 | 2.78863 | 0.002886 |
| **Frontal_Mid_R** | Putamen_R | 37 33 34 | 27 5 2 | 2.69543 | 0.003796 |
| **Frontal_Mid_Orb_L** | Putamen_R | -32 50 -10 | 27 5 2 | 3.154627 | 0.00092 |
| **Frontal_Inf_Oper_L** | Putamen_R | -49 13 19 | 27 5 2 | 2.769493 | 0.003055 |
| **Frontal_Sup_Orb_L** | Pallidum_L | -18 47 -13 | -19 0 0 | 2.924488 | 0.001912 |
| **Frontal_Sup_Orb_R** | Pallidum_L | 17 48 -14 | -19 0 0 | 3.543006 | 0.000243 |
| **Frontal_Sup_Orb_R** | Pallidum_R | 17 48 -14 | 20 0 0 | 3.034144 | 0.001356 |
| **Precentral_L** | Cerebellum_6_L | -40 -6 51 | -23 -59 -22 | 2.779254 | 0.002968 |
| **Rolandic_Oper_R** | Cerebellum_6_R | 52 -6 15 | 25 -58 -24 | 2.85338 | 0.002376 |
| **Frontal_Sup_L** | Cerebellum_7b_L | -19 35 42 | -33 -60 -43 | 2.810573 | 0.002703 |
| **Frontal_Mid_L** | Cerebellum_7b_L | -34 33 35 | -33 -60 -43 | 3.015258 | 0.00144 |
| **Frontal_Sup_L** | Cerebellum_8_L | -19 35 42 | -26 -55 -48 | 2.677109 | 0.004002 |
| **Frontal_Sup_Orb_R** | Cerebellum_9_L | 17 48 -14 | -11 -49 -46 | 2.733556 | 0.003396 |
| **Frontal_Med_Orb_L** | Cerebellum_9_L | -6 54 -7 | -11 -49 -46 | 2.701698 | 0.003727 |
| **Frontal_Med_Orb_R** | Cerebellum_9_L | 7 52 -7 | -11 -49 -46 | 2.667925 | 0.00411 |
| **Precentral_L** | Vermis_4_5 | -40 -6 51 | 1 -52 -6 | 2.73655 | 0.003366 |
| *Hippocampus_R* | *ParaHippocampal_L* | 28 -20 -10 | -22 -16 -21 | 2.607378 | 0.004885 |
| *Hippocampus_L* | *ParaHippocampal_R* | -26 -21 -10 | 24 -15 -20 | 2.713228 | 0.003604 |
| *ParaHippocampal_L* | *Fusiform_R* | -22 -16 -21 | 33 -39 -20 | 2.640001 | 0.004452 |
| *ParaHippocampal_L* | *Heschl_L* | -22 -16 -21 | -43 -19 10 | 2.67281 | 0.004052 |
| *Amygdala_L* | *Heschl_L* | -24 -1 -17 | -43 -19 10 | 3.442398 | 0.000347 |
| *Hippocampus_L* | *Heschl_R* | -26 -21 -10 | 45 -17 10 | 2.923192 | 0.001919 |
| *Hippocampus_R* | *Heschl_R* | 28 -20 -10 | 45 -17 10 | 3.051368 | 0.001284 |
| *ParaHippocampal_L* | *Heschl_R* | -22 -16 -21 | 45 -17 10 | 3.143233 | 0.000954 |
| *ParaHippocampal_R* | *Heschl_R* | 24 -15 -20 | 45 -17 10 | 2.680964 | 0.003958 |
| *Amygdala_L* | *Heschl_R* | -24 -1 -17 | 45 -17 10 | 3.617983 | 0.000185 |
| *Fusiform_L* | *Heschl_R* | -32 -40 -20 | 45 -17 10 | 2.752058 | 0.003216 |
| *Fusiform_R* | *Heschl_R* | 33 -39 -20 | 45 -17 10 | 3.188558 | 0.000823 |
| *Heschl_L* | *Heschl_R* | -43 -19 10 | 45 -17 10 | 2.782393 | 0.00294 |
| *Amygdala_L* | *Temporal_Sup_L* | -24 -1 -17 | -54 -21 7 | 2.966345 | 0.001679 |
| *Heschl_R* | *Temporal_Sup_L* | 45 -17 10 | -54 -21 7 | 2.869956 | 0.00226 |
| *Amygdala_L* | *Temporal_Sup_R* | -24 -1 -17 | 57 -22 7 | 3.132532 | 0.000988 |
| *Heschl_R* | *Temporal_Sup_R* | 45 -17 10 | 57 -22 7 | 2.999887 | 0.001511 |
| *Insula_R* | *Temporal_Pole_Sup_L* | 38 6 2 | -41 15 -20 | 2.824411 | 0.002593 |
| *Heschl_R* | *Temporal_Mid_L* | 45 -17 10 | -57 -34 -2 | 3.299017 | 0.000569 |
| *Temporal_Sup_R* | *Temporal_Mid_L* | 57 -22 7 | -57 -34 -2 | 2.949734 | 0.001768 |
| *Heschl_R* | *Temporal_Mid_R* | 45 -17 10 | 56 -37 -1 | 2.770025 | 0.00305 |
| *Temporal_Mid_L* | *Temporal_Mid_R* | -57 -34 -2 | 56 -37 -1 | 3.522856 | 0.000261 |
| *Heschl_R* | *Temporal_Inf_R* | 45 -17 10 | 53 -31 -22 | 2.931432 | 0.001871 |
| ParaHippocampal_L | Calcarine_L | -22 -16 -21 | -8 -79 6 | 2.712044 | 0.003617 |
| ParaHippocampal_L | Calcarine_R | -22 -16 -21 | 15 -73 9 | 2.749748 | 0.003238 |
| Amygdala_L | Occipital_Mid_L | -24 -1 -17 | -33 -81 16 | 2.601968 | 0.00496 |
| ParaHippocampal_L | Occipital_Mid_R | -22 -16 -21 | 36 -80 19 | 2.704032 | 0.003702 |
| Amygdala_L | Occipital_Mid_R | -24 -1 -17 | 36 -80 19 | 2.778286 | 0.002976 |
| ParaHippocampal_L | Cerebellum_4_5_L | -22 -16 -21 | -15 -43 -17 | 3.19629 | 0.000802 |
| Amygdala_L | Cerebellum_4_5_L | -24 -1 -17 | -15 -43 -17 | 2.691229 | 0.003842 |
| Amygdala_L | Cerebellum_6_L | -24 -1 -17 | -23 -59 -22 | 2.891583 | 0.002115 |
| Heschl_R | Cerebellum_6_L | 45 -17 10 | -23 -59 -22 | 2.816005 | 0.00266 |
| Temporal_Mid_L | Cerebellum_6_L | -57 -34 -2 | -23 -59 -22 | 2.706167 | 0.003679 |
| Insula_R | Cerebellum_6_R | 38 6 2 | 25 -58 -24 | 2.750852 | 0.003228 |
| Amygdala_L | Cerebellum_6_R | -24 -1 -17 | 25 -58 -24 | 2.610102 | 0.004847 |
| Heschl_R | Cerebellum_6_R | 45 -17 10 | 25 -58 -24 | 3.529704 | 0.000255 |
| Temporal_Sup_L | Cerebellum_6_R | -54 -21 7 | 25 -58 -24 | 2.643649 | 0.004406 |
| Temporal_Sup_R | Cerebellum_6_R | 57 -22 7 | 25 -58 -24 | 2.84481 | 0.002439 |
| Heschl_R | Vermis_7 | 45 -17 10 | 1 -72 -25 | 2.763879 | 0.003106 |
| Parietal_Sup_R | Temporal_Inf_L | 25 -59 62 | -51 -28 -23 | 2.71179 | 0.003619 |
| *SupraMarginal_L* | *Angular_L* | -57 -34 30 | -45 -61 36 | 2.650908 | 0.004315 |
| Angular_R | Pallidum_R | 45 -60 39 | 20 0 0 | 2.600855 | 0.004975 |
| Angular_L | Cerebellum_8_L | -45 -61 36 | -26 -55 -48 | 2.776903 | 0.002989 |
| Angular_L | Cerebellum_9_L | -45 -61 36 | -11 -49 -46 | 3.167401 | 0.000882 |
| Angular_R | Cerebellum_9_L | 45 -60 39 | -11 -49 -46 | 2.787784 | 0.002893 |
| Angular_R | Cerebellum_9_R | 45 -60 39 | 9 -49 -46 | 2.685082 | 0.003911 |
| Precuneus_R | Cerebellum_9_R | 9 -56 44 | 9 -49 -46 | 2.76376 | 0.003107 |
| Lingual_L | Heschl_R | -16 -68 -5 | 45 -17 10 | 2.877591 | 0.002208 |
| Lingual_R | Heschl_R | 15 -67 -4 | 45 -17 10 | 2.946722 | 0.001785 |
| Cingulum_Mid_L | Putamen_L | -6 -15 42 | -25 4 2 | 2.854903 | 0.002365 |
| Cingulum_Mid_R | Putamen_L | 7 -9 40 | -25 4 2 | 2.921205 | 0.001931 |
| Cingulum_Mid_L | Putamen_R | -6 -15 42 | 27 5 2 | 2.677698 | 0.003995 |
| Cingulum_Mid_R | Putamen_R | 7 -9 40 | 27 5 2 | 2.675438 | 0.004022 |
| Putamen_L | Heschl_L | -25 4 2 | -43 -19 10 | 2.64912 | 0.004337 |
| Putamen_L | Heschl_R | -25 4 2 | 45 -17 10 | 3.036365 | 0.001347 |
| Pallidum_L | Heschl_R | -19 0 0 | 45 -17 10 | 2.631471 | 0.004562 |
| Putamen_L | Temporal_Sup_L | -25 4 2 | -54 -21 7 | 2.650953 | 0.004315 |
| Putamen_L | Temporal_Sup_R | -25 4 2 | 57 -22 7 | 2.60751 | 0.004883 |
| Putamen_R | Temporal_Inf_L | 27 5 2 | -51 -28 -23 | 2.976984 | 0.001624 |
| *Putamen_R* | *Cerebellum_Crus1_R* | 27 5 2 | 37 -67 -30 | 2.606937 | 0.004891 |
| *Pallidum_L* | *Cerebellum_6_L* | -19 0 0 | -23 -59 -22 | 2.838414 | 0.002486 |
| *Cerebellum_6_L* | *Cerebellum_6_R* | -23 -59 -22 | 25 -58 -24 | 2.848944 | 0.002408 |
| *Putamen_L* | *Cerebellum_7b_L* | -25 4 2 | -33 -60 -43 | 2.642881 | 0.004416 |
| *Pallidum_L* | *Cerebellum_7b_L* | -19 0 0 | -33 -60 -43 | 2.848798 | 0.002409 |
| *Cerebellum_Crus1_R* | *Cerebellum_9_L* | 37 -67 -30 | -11 -49 -46 | 2.959981 | 0.001712 |
| *Cerebellum_Crus2_R* | *Cerebellum_9_L* | 32 -69 -40 | -11 -49 -46 | 3.330639 | 0.000511 |
| *Cerebellum_7b_L* | *Cerebellum_9_L* | -33 -60 -43 | -11 -49 -46 | 2.691941 | 0.003834 |
| *Cerebellum_7b_R* | *Cerebellum_9_L* | 35 -64 -47 | -11 -49 -46 | 2.721479 | 0.003518 |
| *Cerebellum_8_L* | *Cerebellum_9_L* | -26 -55 -48 | -11 -49 -46 | 2.61161 | 0.004826 |
| *Putamen_L* | *Vermis_3* | -25 4 2 | 1 -40 -11 | 2.679493 | 0.003975 |
| *Putamen_R* | *Vermis_3* | 27 5 2 | 1 -40 -11 | 2.674559 | 0.004032 |
| *Pallidum_L* | *Vermis_3* | -19 0 0 | 1 -40 -11 | 2.646315 | 0.004372 |
| *Pallidum_R* | *Vermis_3* | 20 0 0 | 1 -40 -11 | 2.623737 | 0.004663 |
| *Cerebellum_8_L* | *Vermis_7* | -26 -55 -48 | 1 -72 -25 | 2.602372 | 0.004954 |
| *Cerebellum_9_L* | *Vermis_7* | -11 -49 -46 | 1 -72 -25 | 2.857837 | 0.002344 |
| *Cerebellum_9_R* | *Vermis_7* | 9 -49 -46 | 1 -72 -25 | 2.615067 | 0.004779 |

**Diffusion tensor imaging:**

| Brain node 1* | Brain node 2* | MNI coordinate 1# | MNI coordinate 2# | t-value | p-value |
| --- | --- | --- | --- | --- | --- |
| ***Frontal_Sup_Orb_R*** | ***Frontal_Mid_R*** | 17 48 -14 | 37 33 34 | 2.948183 | 0.001734 |
| ***Frontal_Mid_R*** | ***Frontal_Mid_Orb_R*** | 37 33 34 | 32 53 -11 | 2.965526 | 0.001642 |
| **Frontal_Sup_Orb_R** | Hippocampus_R | 17 48 -14 | 28 -20 -10 | 3.043932 | 0.00128 |
| **Frontal_Sup_R** | Heschl_R | 20 31 44 | 45 -17 10 | 2.815486 | 0.00261 |
| **Frontal_Sup_R** | Temporal_Mid_L | 20 31 44 | -57 -34 -2 | 2.772786 | 0.002967 |
| **Frontal_Mid_R** | Temporal_Mid_L | 37 33 34 | -57 -34 -2 | 2.912428 | 0.001939 |
| **Frontal_Sup_R** | Temporal_Inf_L | 20 31 44 | -51 -28 -23 | 3.001188 | 0.001467 |
| **Frontal_Inf_Tri_R** | Temporal_Inf_L | 49 30 14 | -51 -28 -23 | 3.090496 | 0.001101 |
| **Frontal_Inf_Tri_L** | Precuneus_L | -47 30 14 | -8 -56 48 | 2.726434 | 0.003405 |
| **Frontal_Mid_L** | Cuneus_R | -34 33 35 | 13 -79 28 | 3.061236 | 0.00121 |
| **Frontal_Med_Orb_L** | Cuneus_R | -6 54 -7 | 13 -79 28 | 2.616205 | 0.00469 |
| **Frontal_Inf_Tri_R** | Occipital_Sup_L | 49 30 14 | -18 -84 28 | 2.632934 | 0.00447 |
| **Frontal_Inf_Tri_R** | Occipital_Mid_L | 49 30 14 | -33 -81 16 | 2.655832 | 0.004185 |
| **Frontal_Sup_Orb_L** | Occipital_Inf_L | -18 47 -13 | -37 -78 -8 | 2.68464 | 0.003849 |
| **Rectus_L** | Occipital_Inf_L | -6 37 -18 | -37 -78 -8 | 2.606679 | 0.004819 |
| **Frontal_Sup_R** | Cingulum_Mid_R | 20 31 44 | 7 -9 40 | 2.756724 | 0.003113 |
| **Frontal_Mid_R** | Cingulum_Mid_R | 37 33 34 | 7 -9 40 | 2.753009 | 0.003148 |
| **Frontal_Inf_Tri_R** | Thalamus_L | 49 30 14 | -12 -18 8 | 2.605123 | 0.00484 |
| **Frontal_Sup_Orb_R** | Cerebellum_3_R | 17 48 -14 | 12 -34 -19 | 2.907542 | 0.001969 |
| **Frontal_Inf_Tri_R** | Cerebellum_7b_L | 49 30 14 | -33 -60 -43 | 2.864093 | 0.002251 |
| **Frontal_Inf_Tri_R** | Vermis_7 | 49 30 14 | 1 -72 -25 | 2.692651 | 0.00376 |
| Hippocampus_L | Pallidum_R | -26 -21 -10 | 20 0 0 | 3.457133 | 0.000316 |
| Hippocampus_L | Thalamus_R | -26 -21 -10 | 12 -18 8 | 2.87939 | 0.002148 |
| Hippocampus_R | Vermis_7 | 28 -20 -10 | 1 -72 -25 | 3.164489 | 0.000863 |
| Precuneus_L | Temporal_Sup_L | -8 -56 48 | -54 -21 7 | 3.314631 | 0.00052 |
| Precuneus_L | Temporal_Pole_Sup_L | -8 -56 48 | -41 15 -20 | 3.140185 | 0.000936 |
| Parietal_Sup_L | Temporal_Mid_L | -24 -60 59 | -57 -34 -2 | 2.994856 | 0.001497 |
| Precuneus_L | Temporal_Mid_L | -8 -56 48 | -57 -34 -2 | 3.015499 | 0.001402 |
| Parietal_Sup_L | Temporal_Inf_L | -24 -60 59 | -51 -28 -23 | 2.681395 | 0.003886 |
| Precuneus_L | Pallidum_R | -8 -56 48 | 20 0 0 | 3.430424 | 0.000347 |
| SupraMarginal_R | Cerebellum_Crus1_L | 57 -32 34 | -36 -67 -29 | 2.737971 | 0.003291 |
| Precuneus_L | Cerebellum_3_R | -8 -56 48 | 12 -34 -19 | 2.832031 | 0.002482 |
| Calcarine_L | Temporal_Sup_R | -8 -79 6 | 57 -22 7 | 3.047353 | 0.001266 |
| Cuneus_R | Parietal_Inf_L | 13 -79 28 | -44 -46 47 | 2.654728 | 0.004198 |
| Occipital_Mid_L | Parietal_Inf_L | -33 -81 16 | -44 -46 47 | 2.630423 | 0.004503 |
| *Calcarine_L* | *Occipital_Mid_L* | -8 -79 6 | -33 -81 16 | 2.911814 | 0.001943 |
| *Calcarine_L* | *Occipital_Inf_L* | -8 -79 6 | -37 -78 -8 | 2.765985 | 0.003028 |
| Cuneus_L | Thalamus_L | -7 -80 27 | -12 -18 8 | 3.044983 | 0.001275 |
| Occipital_Mid_L | Cerebellum_10_L | -33 -81 16 | -23 -34 -42 | 2.664906 | 0.004076 |
| Calcarine_L | Vermis_7 | -8 -79 6 | 1 -72 -25 | 3.445874 | 0.000329 |
| Cingulum_Post_R | Calcarine_L | 6 -42 22 | -8 -79 6 | 2.774792 | 0.00295 |
| *Pallidum_L* | *Thalamus_L* | -19 0 0 | -12 -18 8 | 3.133992 | 0.000955 |
| *Thalamus_R* | *Cerebellum_Crus1_L* | 12 -18 8 | -36 -67 -29 | 2.981882 | 0.00156 |
| *Pallidum_R* | *Cerebellum_Crus1_R* | 20 0 0 | 37 -67 -30 | 2.719439 | 0.003476 |
| *Cerebellum_6_R* | *Cerebellum_7b_L* | 25 -58 -24 | -33 -60 -43 | 3.289062 | 0.000568 |
| *Cerebellum_6_R* | *Cerebellum_9_L* | 25 -58 -24 | -11 -49 -46 | 2.692418 | 0.003763 |
| *Cerebellum_Crus1_R* | *Cerebellum_10_L* | 37 -67 -30 | -23 -34 -42 | 2.785143 | 0.00286 |
| *Cerebellum_7b_R* | *Vermis_7* | 35 -64 -47 | 1 -72 -25 | 2.768849 | 0.003003 |

**References**

Callicott, J., Egan, M. F., Mattay, V., Bertolino, A., Bone, A. D., Verchinski, B. A., & Weinberger, D. R. (2004). Abnormal fMRI response of the dorsolateral prefrontal cortex in cognitively intact siblings of patients with schizophrenia. *Am. J. Psychiatry, 160*, 709-719.

Cao, H., Plichta, M. M., Schafer, A., Haddad, L., Grimm, O., Schneider, M., . . . Tost, H. (2014). Test-retest reliability of fMRI-based graph theoretical properties during working memory, emotion processing, and resting state. *Neuroimage, 84*, 888-900. doi:10.1016/j.neuroimage.2013.09.013

Guo, G., Ou, X. M., Roettger, M., & Shih, J. C. (2008). The VNTR 2 repeat in MAOA and delinquent behavior in adolescence and young adulthood: associations and MAOA promoter activity. *Eur. J. Hum. Genet., 16*, 626-634. doi:10.1038/sj.ejhg.5201999

Hariri, A. R., Mattay, V. S., Tessitore, A., Kolachana, B., Fera, F., Goldman, D., . . . Weinberger, D. R. (2002). Serotonin transporter genetic variation and the response of the human amygdala. *Science, 297*, 400-403. doi:10.1126/science.1071829

Sabol, S. Z., Hu, S., & Hamer, D. (1998). A functional polymorphism in the monoamine oxidase A gene promoter. *Hum. Genet., 103*, 273-279.

Tzourio-Mazoyer, N., Landeau, B., Papathanassiou, D., Crivello, F., Etard, O., Delcroix, N., . . . Joliot, M. (2002). Automated anatomical labeling of activations in SPM using a macroscopic anatomical parcellation of the MNI MRI single-subject brain. *Neuroimage, 15*, 273-289. doi:10.1006/nimg.2001.0978
